# Empagliflozin Use Is Associated With Lower Risk of All-Cause Mortality, Hospitalization for Heart Failure, and End-Stage Renal Disease Compared to DPP-4i in Nordic Type 2 Diabetes Patients: Results From the EMPRISE (Empagliflozin Comparative Effectiveness and Safety) Study

**DOI:** 10.1155/2024/6142211

**Published:** 2024-10-12

**Authors:** Gisle Langslet, Thomas Nyström, Dorte Vistisen, Bendix Carstensen, Emilie Toresson Grip, Paula Casajust, Giorgi Tskhvarashvili, Fabian Hoti, Riho Klement, Kristina Karlsdotter, Mikko Tuovinen, Anne Pernille Ofstad, Maria Lajer, Christina Shay, Lisette Koeneman, Soulmaz Fazeli Farsani, Leo Niskanen, Sigrun Halvorsen

**Affiliations:** ^1^Lipid Clinic, Department of Endocrinology, Morbid Obesity and Preventive Medicine, Oslo University Hospital 0424, Oslo, Norway; ^2^Department of Clinical Science and Education, Södersjukhuset, Karolinska Institutet 118 83, Stockholm, Sweden; ^3^Clinical Epidemiology, Steno Diabetes Center Copenhagen 2730, Herlev, Denmark; ^4^A/I and Analytics, Novo Nordisk A/S, AI and Analytics, Søborg, Denmark; ^5^Real World Data and Data Analytics, Quantify Research 11221, Stockholm, Sweden; ^6^Department of Medicine, Karolinska Institutet 141 57, Huddinge, Sweden; ^7^Real-World Data and Data Analytics, TFS HealthScience 08007, Barcelona, Spain; ^8^Global Database Studies, Real World Solutions, IQVIA 10119, Tallinn, Estonia; ^9^Global Database Studies, Real World Solutions, IQVIA 02130, Espoo, Finland; ^10^Global Database Studies, Real World Solutions, IQVIA, 51013, Tartu, Estonia; ^11^Market Access, Boehringer Ingelheim Pharmaceuticals 12032, Stockholm, Sweden; ^12^Global Medical Affairs, Boehringer Ingelheim Pharmaceuticals 00180, Helsinki, Finland; ^13^Type 2 Diabetes and Metabolism, Medical Department, Boehringer Ingelheim 1383, Oslo, KS, Norway; ^14^Department of Medicine, Oslo Diabetes Research Center 0424, Oslo, Norway; ^15^Global Integrated Evidence, Boehringer Ingelheim Pharmaceuticals 2100, Copenhagen, Denmark; ^16^Global Integrated Evidence, Boehringer Ingelheim Pharmaceuticals, Inc. 06877, Ridgefield, Connecticut, USA; ^17^Diabetes Global Medical Affairs, Lilly Deutschland GmbH 61352, Bad Homburg, Germany; ^18^Global Integrated Evidence, Boehringer Ingelheim International GmbH 55216, Ingelheim, Germany; ^19^Päijät-Häme Joint Authority for Health and Wellbeing, Päijät-Häme Central Hospital 15850, Lahti, Finland; ^20^Institute of Clinical Medicine, University of Eastern Finland 70210, Kuopio, Finland; ^21^Department of Cardiology, Oslo University Hospital Ullevål 0450, Oslo, Norway; ^22^Institute of Clinical Medicine, University of Oslo 0372, Oslo, Norway

**Keywords:** cardiovascular diseases, comparative effectiveness, dipeptidyl peptidase-4 inhibitors, empagliflozin, end-stage renal disease, heart failure, sodium-glucose cotransporter-2 inhibitors, type 2 diabetes mellitus

## Abstract

**Objective**: To evaluate the effectiveness of empagliflozin in reducing all-cause mortality (ACM), hospitalization for heart failure (HHF), myocardial infarction (MI), stroke, cardiovascular mortality (CVM), and end-stage renal disease (ESRD) in routine clinical practice in the Nordic countries of the Empagliflozin Comparative Effectiveness and Safety (EMPRISE) study.

**Methods**: This noninterventional, multicountry cohort study used secondary data from four Nordic countries (Denmark, Sweden, Finland, and Norway). Propensity score (PS) matched (1:1) adults with type 2 diabetes (T2D) initiating empagliflozin (a sodium-glucose cotransporter-2 inhibitor) during 2014–2018 who were compared to those initiating a dipeptidyl peptidase-4 inhibitor (DPP-4i). Cox proportional hazards regression modelling was used to assess the risk for ACM, HHF, MI, stroke, CVM, and ESRD. Meta-analyses were conducted and hazard ratios (HRs) with 95% confidence intervals (CIs) from random-effects models were calculated.

**Results**: A total of 43,695 pairs of PS-matched patients were identified. Patients initiating empagliflozin exhibited a 49% significantly lower risk of ACM (HR: 0.51, 95% CI 0.40–0.64) compared to DPP-4i. Additionally, empagliflozin was associated with a 36% significantly lower risk of HHF (HR: 0.64, 95% CI 0.46–0.89), a 52% significantly lower risk of CVM (HR: 0.48, 95% CI 0.37–0.63), and a 66% significantly lower risk of ESRD (HR: 0.34, 95% CI 0.15–0.77) compared to DPP-4i. No significant differences were observed in the risk of stroke and MI between patients initiating empagliflozin compared with those initiating a DPP-4i. Results were generally consistent for subgroups (with/without pre-existing CV disease or congestive heart failure) and in sensitivity analyses.

**Conclusion**: Empagliflozin initiation was associated with a significantly reduced risk of ACM, HHF, CVM, and ESRD compared with initiation of DPP-4i in patients with T2D when examining routine clinical practice data from Nordic countries.

**Summary**

• The EMPA-REG OUTCOME clinical trial demonstrated that empagliflozin benefits the heart and kidneys and has a positive metabolic impact in patients with type 2 diabetes and established cardiovascular disease. However, less is known about the real-world benefits of empagliflozin in patients without established CV disease or heart failure in Nordic populations.
• The EMPRISE (Empagliflozin Comparative Effectiveness and Safety) Europe and Asia study is a noninterventional, cohort study in 11 countries that included secondary data from four Nordic countries (Denmark, Sweden, Finland, and Norway). Propensity score matched (1:1) adults with type 2 diabetes initiating empagliflozin (a sodium-glucose cotransporter-2 inhibitor) during 2014–2018 who were compared to those initiating a dipeptidyl peptidase-4 inhibitor (DPP-4i).
• Among the 43,695 pairs of PS-matched patients identified across the four Nordic countries, patients initiating empagliflozin exhibited a 49% significantly lower risk of all-cause mortality, 36% significantly lower risk of hospitalization for heart failure, 52% significantly lower risk of cardiovascular mortality, and a 66% significantly lower risk of incident end-stage renal disease compared to patients initiating DPP-4i. No significant differences in risk were observed for stroke and myocardial infarction between patients initiating empagliflozin compared with those initiating DPP-4i.
• Results were generally consistent among patients with/without pre-existing CV disease or congestive heart failure.

## 1. Introduction

Diabetes mellitus is a significant public health concern that is approaching epidemic proportions worldwide [1]. According to the 2021 International Diabetes Federation estimates, the number of adult patients with diabetes (among ages 20–79 years) will rise from 61.4 million in 2021 to 69.2 million by 2045 [2]. Patients with type 2 diabetes (T2D) have an increased risk of developing several comorbidities, including cardiovascular (CV) and renal diseases [3]. Atherosclerosis, coronary heart disease, heart failure (HF), angina, myocardial infarction (MI), and stroke are significant CV events that can affect up to one-third of patients with T2D. Furthermore, approximately 50% of patients with T2D show evidence of chronic kidney disease [3, 4].

Sodium-glucose cotransporter-2 inhibitors (SGLT2i) are among the most recent medications for the treatment of T2D. Currently, metformin monotherapy is still commonly prescribed as the first line of glucose-lowering therapy in clinical practice. In Nordic countries, SGLT2i is typically employed as a second-line glucose-lowering therapy [5]. Empagliflozin is one of the approved SGLT2i that reduces hyperglycemia by decreasing renal reabsorption of glucose thereby increasing glycosuria [6, 7]. [1, 2] The pivotal EMPA-REG OUTCOME [8, 9] and EMPA-KIDNEY [10] trials demonstrated that adding empagliflozin to the standard of care benefits the heart and kidneys in addition to having a positive metabolic impact in patients with T2D and established CV disease. These findings were also observed among patients with HF with reduced or preserved ejection fraction in the EMPEROR-Reduced [11] and EMPEROR-Preserved trials [12], respectively. The initial EMPRISE (Empagliflozin Comparative Effectiveness and Safety) study included patients with T2D from the United States and demonstrated in routine clinical care settings that empagliflozin was associated with a significantly lower risk of hospitalization for heart failure (HHF), all-cause mortality (ACM), cardiovascular mortality (CVM), a composite of MI, stroke, and ACM, when compared with sitagliptin and other dipeptidyl peptidase-4 inhibitors (DPP-4i) [13–15].

Despite the strong available evidence of the beneficial effects of empagliflozin, a need still exists to assess the effectiveness and safety of empagliflozin in additional real-world settings across diverse regional populations. Overall, the proportion of empagliflozin use in the studies investigating any SGLT2i has been < 10% [16–18], and, therefore, the results may not be generalizable to patients initiating specifically empagliflozin therapy. Therefore, the aim of this EMPRISE (Empagliflozin Comparative Effectiveness and Safety) study was to evaluate the effectiveness of empagliflozin in reducing ACM, HHF, MI, stroke, CVM, and end-stage renal disease (ESRD) in routine clinical practice in the Nordic countries with comprehensive registers and homogenous patient populations.

## 2. Methods

This comparative, noninterventional, multicountry cohort study analyzed pseudonymized register-based data of patients with a diagnosis of T2D and treatment of either empagliflozin or DPP-4i from four Nordic countries (Denmark, Finland, Norway, and Sweden).

All data were obtained electronically and recorded from longitudinal secondary data sources at the national and regional level, separately in each country without an access to the individual-level data at any time of the study. Thus, no ethical approval or informed consent was required in any of the countries. However, applications to access and use data were approved for main data holders (Sweden: The Swedish Ethical Review Authority (Etikprövningsmyndigheten) Reference ID: 2018/2335-31; Finland: Finnish Institute for Health and Welfare (Terveyden ja hyvinvoinnin laitos) Reference ID: THL/143/5.05.00/2019l; Norway: REK (Regionale Komiteer For Medisinsk og Helsefaglig Forskningsetikk) Reference ID: 2019/373 REK sør-øst B; and Denmark: access and use of the described data were approved by the Danish Data Protection Agency (j-No. VD-2019-197) and the Danish Patient Safety Authority (j-No. 3-3013-2959/1)). Patient registers, prescription registers, and cause of death registers were used across the Nordic countries, virtually covering 100% of the population [19]. Patients with dispensations of empagliflozin or any DPP-4i were identified in the prescription registers and then linked to the other registers used in this study. Specifications of the data sources used in this study can be found in Table S1.

Eligible patients were aged ≥ 18 years at the first prescription of empagliflozin or DPP-4i and had a diagnosis of T2D (based on codes from the 10^th^ revision of the International Classification of Diseases and Related Health Problems (ICD-10) [20] or at least one previous prescription of metformin). Patients with type 1 diabetes, gestational diabetes, or other types of diabetes mellitus (including diabetes mellitus secondary to endocrinopathies or diseases of the exocrine pancreas) at any time before the index date (ID) were excluded. Other exclusion criteria included any diagnosis of ESRD before the ID, less than 12 months of available data before the ID, incomplete history of drug dispensations/other records of drug use, and/or missing/ambiguous data on age or sex.

**Figure 1:**
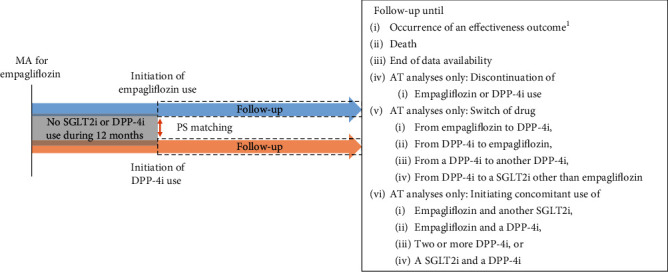
Overview of the study periods. AT = as-treated; DPP-4i = dipeptidyl peptidase-4 inhibitor; MA = marketing authorization; PS = propensity score; SGLT2i = sodium-glucose cotransporter-2 inhibitor. ^1^In analyses investigating effectiveness outcomes, the occurrence of the outcome in question was observed until the end of the follow-up (e.g., while investigating hospitalization for heart failure, the follow-up did not end at the occurrence of a stroke).

All individuals initiating either empagliflozin or DPP-4i between the market authorization date of empagliflozin (May 2014 onwards) and the end of data availability (December 2018 at the latest; new users) were selected in each country (Figure 1). Patients with concomitant use of an SGLT2i and a DPP-4i were censored.

The main exposure in this study is the initiation of empagliflozin (Anatomical Therapeutic Chemical code (ATC) codes: A10BK03 (also A10BX12 refers to empagliflozin in Finland), A10BD20 refers to the combination of empagliflozin and metformin). Initiation of any DPP-4i was the main comparator in the analyses. Exposure period was assumed to begin on the date of a dispensation. A supply, which indicated the duration of exposure after a dispensation, was defined for each dispensation based on the amount purchased. Definitions for the calculation of exposure periods can be found in the Supporting Information section.

Patients in the empagliflozin and DPP-4i cohorts underwent 1:1 propensity score (PS) matching based on ≥ 105 covariates (demographics, burden of comorbidities, diabetes-related complications, diabetes medications, lifestyle factors, prior healthcare utilization, and laboratory test results) in each database. Definitions and full list of covariates can be found in Table S2. Postmatching covariate balance was assessed by absolute standardized differences (ASD) with ASD > 0.1 considered to be a meaningful imbalance.

For all PS-matched patients, the follow-up began on the ID and continued in an “as-treated” (AT) approach until one of the following events occurred: outcome, death, discontinuation of the initial drug, switch to or initiation of concomitant use with another study drug (empagliflozin, any SGLT2i, any DPP-4i), or end of data availability. The minimum follow-up time was 1 day for each patient who contributed to the outcome analyses, and the follow-up time contributing to the analyses started 1 day after the ID. Each patient was included only once in each subcohort.

Primary effectiveness outcomes included ACM, HHF, MI, and stroke. ACM was defined as any death registered in the respective cause of death registry for each country. HHF was defined as primary diagnosis of HF associated with hospital admission. MI and stroke were defined as any primary diagnosis associated with hospital admission. The secondary effectiveness outcomes were CVM and ESRD. CVM was defined as a death from any CV condition, death from diabetes with vascular complication, or death within 30 days of a CV event-related hospitalization. ESRD was defined as at least 1 ESRD-specific diagnosis/procedure/laboratory measurement associated with healthcare encounters, including hospitalizations and specialist outpatient encounters. The full definitions for study outcomes can be found in Table S4.

Comparisons were performed between patients initiating empagliflozin and those initiating any DPP-4i. DPP-4i was used as a comparator, as it is considered the same line of treatment as empagliflozin in all countries, thus comparing patients in a similar phase of the disease.

Sensitivity analyses for the primary outcomes were performed using the intention-to-treat (ITT) approach. In the ITT approach, follow-up continued until the occurrence of outcome, death, or end of data availability regardless of changes in drug treatment. Subgroup analyses were performed focusing on patients with and without the following conditions: pre-existing congestive heart failure (CHF) and history of CV disease at any time prior to the ID (look-back period was since 2005).

Continuous covariates were described by mean, standard deviation (SD), median, 25^th^ and 75^th^ percentiles, minimum, and maximum. Categorical covariates were described by proportion and frequency in each category.

For the primary outcomes, incidence rates (events per 1,000 person-years with corresponding 95% confidence intervals (CIs)) were calculated separately for each subcohort. Adjusted hazard ratios (HRs) with 95% CIs were estimated using Cox proportional hazard models adjusted for unbalanced PS variables at baseline. The meta-analysis was performed for the primary and secondary outcomes to combine individual country-level results by using random effect meta-analysis models. It is important to note that numbers in the text refer to the random-effects model if not otherwise specified. R software (version 3.5.0) was used for data management, statistical analyses, and graphics for the meta-analyses. Heterogeneity (*I*^2^) in the effect size was estimated across countries (0%–40%: may not be important; 30%–60%: may represent moderate heterogeneity; 50%–90%: may represent substantial heterogeneity; 75%–100%: considerable heterogeneity) [21, 22].

## 3. Results

### 3.1. Study Population

**Figure 2:**
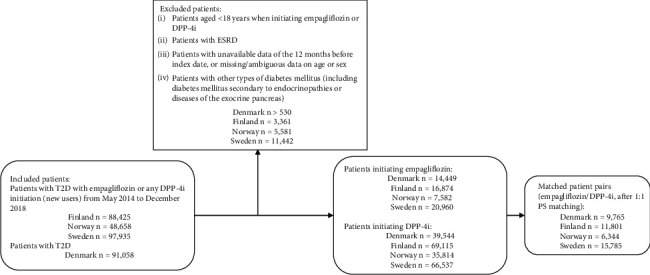
Attrition flowchart for four countries from inclusion to propensity score matched pairs. DPP-4i = dipeptidyl peptidase-4 inhibitor; ESRD = end-stage renal disease; PS = propensity score; SGLT2i = sodium-glucose cotransporter-2 inhibitor; T2D = type 2 diabetes.

The main study population consisted of 43,695 empagliflozin/DPP-4i PS-matched patient pairs in total (9765 pairs in Denmark, 11,801 in Finland, 6344 in Norway, and 15,785 in Sweden) after applying the eligibility criteria and performing the PS matching (Figure 2). The overall mean follow-up was 0.7 years. The mean follow-up time was similar between empagliflozin (0.54–1.01 years) and DPP-4i (0.81–1.04 years) initiators across countries and outcomes.

Baseline characteristics were similar in each country after performing PS-matching and comparable between empagliflozin and DPP-4i initiators (ASD < 0.1) (Table S5). Overall, mean age was approximately 62 years, and the majority of patients (~60%) were male. The most common comorbidities were hyperlipidemia and hypertension (~15% and ~25% of patients, respectively). The proportion of patients diagnosed with CHF was low (~6%). The cohort from Sweden was comparatively older (mean age~63 years). Prevalence of ischemic heart disease was higher in Sweden (~22%) and Norway (~21%) than in Denmark (~10%) and Finland (~15%).

### 3.2. Primary Outcomes

#### 3.2.1. ACM

**Figure 3:**
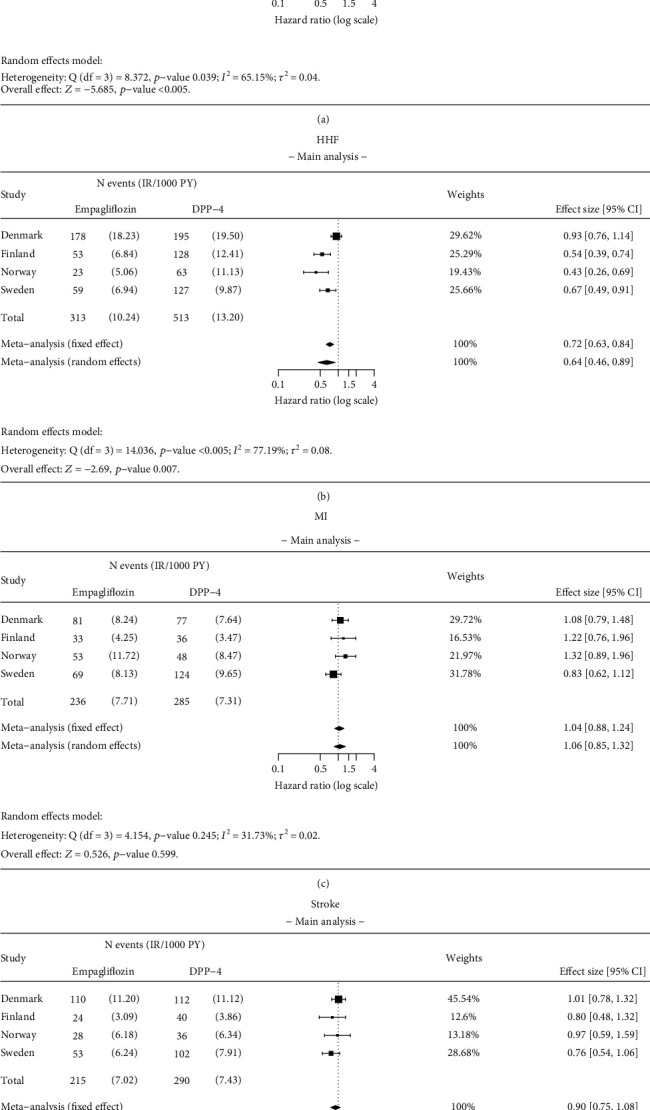
Results of the meta-analysis for (a) all-cause mortality (ACM), (b) hospitalization for heart failure (HHF), (c) myocardial infarction (MI), and (d) stroke. Analysis details: As-treated (AT). Numbers < 5 are not shown due to data protection, but they are included in meta-analysis. If values < 5 exist, a total number of events and incidence rates are presented as intervals.

Initiation of empagliflozin was associated with a 49% significantly lower risk of ACM compared with DPP-4i (overall HR 0.51; 95% CI 0.40–0.64) (Figure 3(a)). This association was comparable among countries (HR 0.58; 95% CI 0.47–0.71 in Denmark; 0.36; 95% CI 0.27–0.48 in Finland; 0.62; 95% CI 0.42–0.90 in Norway; and 0.53; 95% CI 0.41–0.68 in Sweden; *I*^2^ = 65.15%).

#### 3.2.2. HHF

Initiation of empagliflozin was associated with a 36% significantly lower risk of HHF compared with DPP-4i initiators (overall HR 0.64; 95% CI 0.46–0.89) (Figure 3(b)). The strength of this association varied across the countries (HR 0.93; 95% CI 0.76–1.14 in Denmark; 0.54; 95% CI 0.39–0.74 in Finland; 0.43; 95% CI 0.26–0.69 in Norway; and 0.67; 95% CI 0.49–0.91 in Sweden; *I*^2^ = 77.19%).

#### 3.2.3. MI

No significant difference was observed in the risk of MI between patients receiving empagliflozin and DPP-4i initiators (overall HR 1.06; 95% CI 0.85–1.32) (Figure 3(c)). This result was similar across the countries (HR 1.08; 95% CI 0.79–1.48 in Denmark; 1.22; 95% CI 0.76–1.96 in Finland; 1.32; 95% CI 0.89–1.96 in Norway; and 0.83; 95% CI 0.62–1.12 in Sweden; *I*^2^ = 31.73%).

#### 3.2.4. Stroke

No significant difference was observed in the risk of stroke between patients receiving empagliflozin compared with DPP-4i initiators (overall HR 0.90; 95% CI 0.75–1.08) (Figure 3(d)). This result was similar across countries (HR 1.01; 95% CI 0.78–1.32 in Denmark; 0.80; 95% CI 0.48–1.32 in Finland; 0.97; 95% CI 0.59–1.59 in Norway; and 0.76; 95% CI 0.54–1.06 in Sweden; *I*^2^ = 1.47%).

### 3.3. Secondary Outcomes

#### 3.3.1. CVM

**Figure 4:**
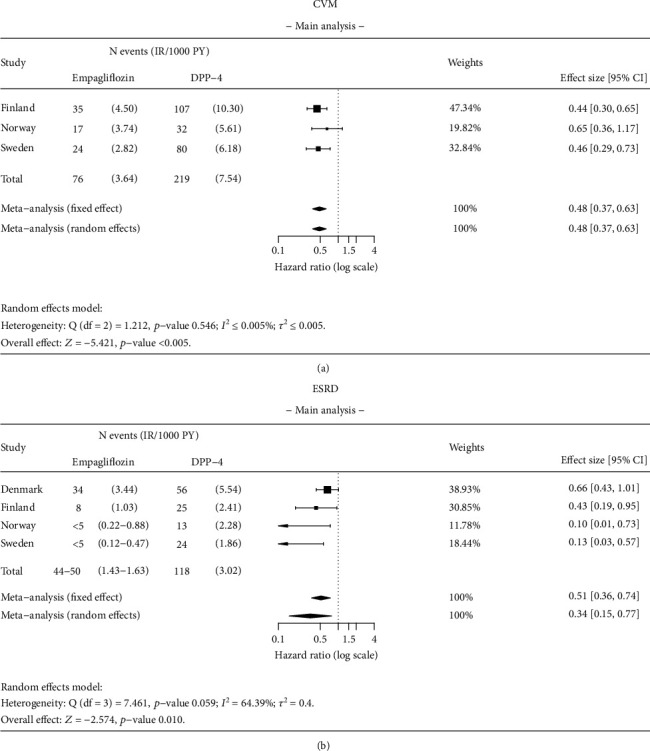
Results of the meta-analysis for (a) cardiovascular mortality (CVM) and (b) end-stage renal disease (ESRD). Analysis details: As-treated (AT). Numbers < 5 are not shown due to data protection, but they are included in meta-analysis. If values < 5 exist, a total number of events and incidence rates are presented as intervals.

Initiation of empagliflozin was associated with a 52% significantly lower risk of CVM compared with DPP-4i initiators (overall HR 0.48; 95% CI 0.37–0.63) (Figure 4(a)). The strength of this association varied across the countries (HR 0.44; 95% CI 0.30–0.65 in Finland; 0.65; 95% CI 0.36–1.17 in Norway; and 0.46; 95% CI 0.29–0.73 in Sweden; I^2^ ≤ 0.005%).

#### 3.3.2. ESRD

Initiation of empagliflozin was associated with a 66% significantly lower risk of ESRD compared with DPP-4i initiators (overall HR 0.34; 95% CI 0.15–0.77) (Figure 4(b)). The strength of this association varied across the countries (HR 0.66; 95% CI 0.43–1.01 in Denmark, 0.43; 95% CI 0.19–0.95 in Finland; 0.10; 95% CI 0.01–0.73 in Norway; and 0.13; 95% CI 0.03–0.57 in Sweden; *I*^2^ = 64.39%).

### 3.4. Subgroup Analyses

**Figure 5:**
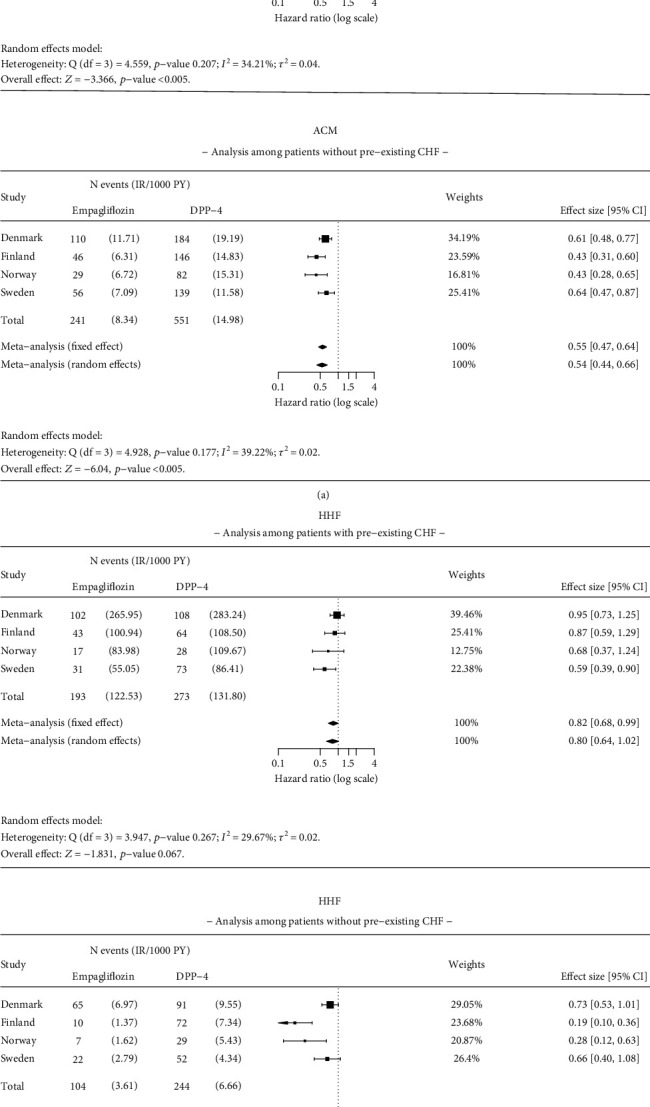
Results of the meta-analysis for (a) all-cause mortality (ACM) and (b) hospitalization for heart failure (HHF) among patients with and without pre-existing congestive heart failure (CHF). Analysis details: As-treated (AT). Numbers < 5 are not shown due to data protection, but they are included in meta-analysis. If values < 5 exist, a total number of events and incidence rates are presented as intervals. The time window for CHF is ever before the index date.

**Figure 6:**
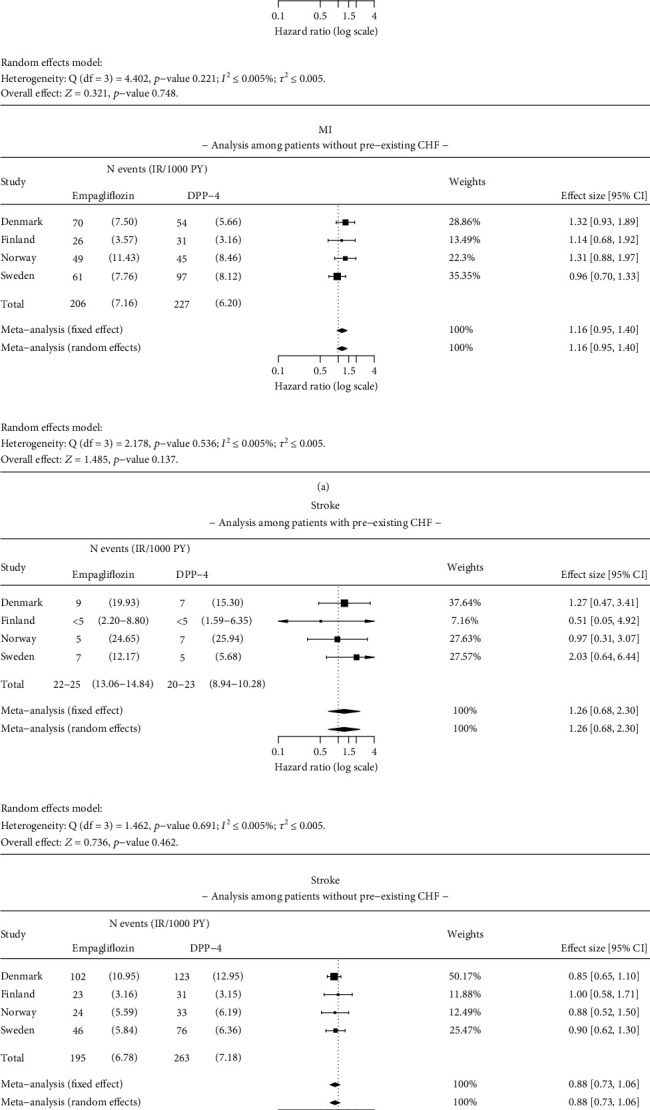
Results of the meta-analysis for (a) myocardial infarction (MI) and (b) stroke among patients with and without pre-existing congestive heart failure (CHF). Analysis details: As-treated (AT). Numbers < 5 are not shown due to data protection, but they are included in meta-analysis. If values < 5 exist, a total number of events and incidence rates are presented as intervals. The time window for CHF is ever before the index date.

Results were consistent with the main analysis for ACM, MI, and stroke in patients with and without pre-existing CHF at baseline (Figures 5 and 6). The HR for ACM was 0.55 (95% CI 0.38–0.78) in patients with CHF and 0.54 (95% CI 0.44–0.66) in patients without pre-existing CHF at baseline (Figure 5(a)). A significantly lower (58%) risk of HHF was found in patients without pre-existing CHF at baseline (HR 0.42 95% CI 0.22–0.81) (Figure 5(b)). No statistically significant difference was observed in the risk of HHF in patients with pre-existing CHF (HR 0.80; 95% CI 0.64–1.02) between empagliflozin and DPP-4i initiators (Figure 5(b)).

**Figure 7:**
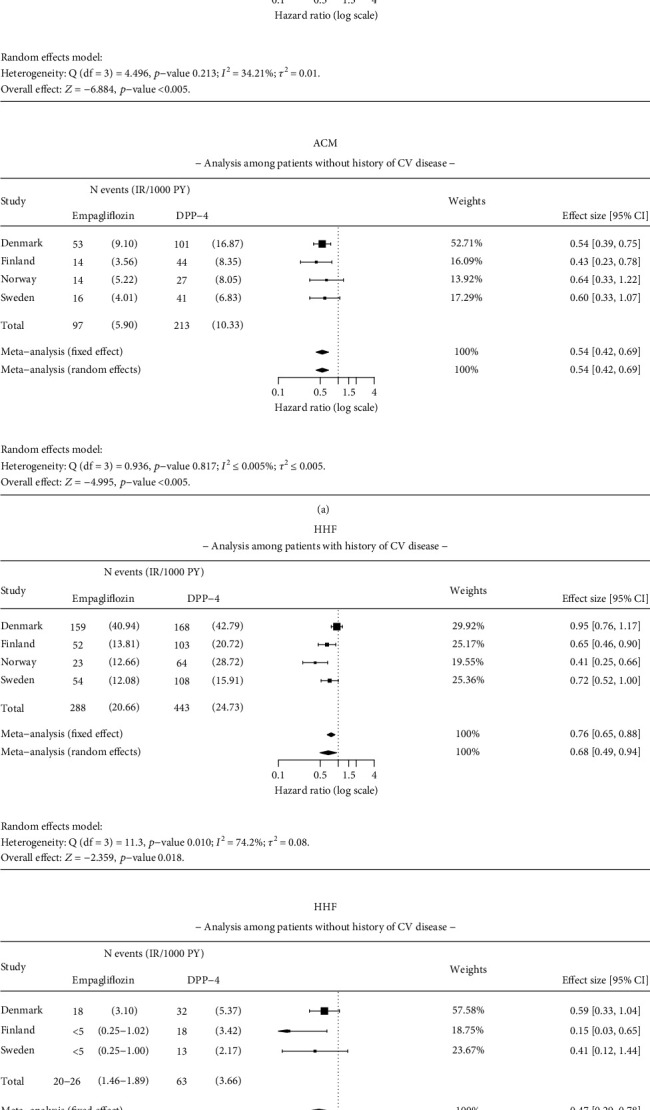
Results of the meta-analysis for (a) all-cause mortality (ACM) and (b) hospitalization for heart failure (HHF) among patients with and without pre-existing cardiovascular (CV) disease. Analysis details: As-treated (AT). Country-level results with insufficient number of events for analysis in either of the study groups are omitted from the analysis. Numbers < 5 are not shown due to data protection, but they are included in meta-analysis. If values < 5 exist, a total number of events and incidence rates are presented as intervals. The time window for CV disease is ever before the index date.

**Figure 8:**
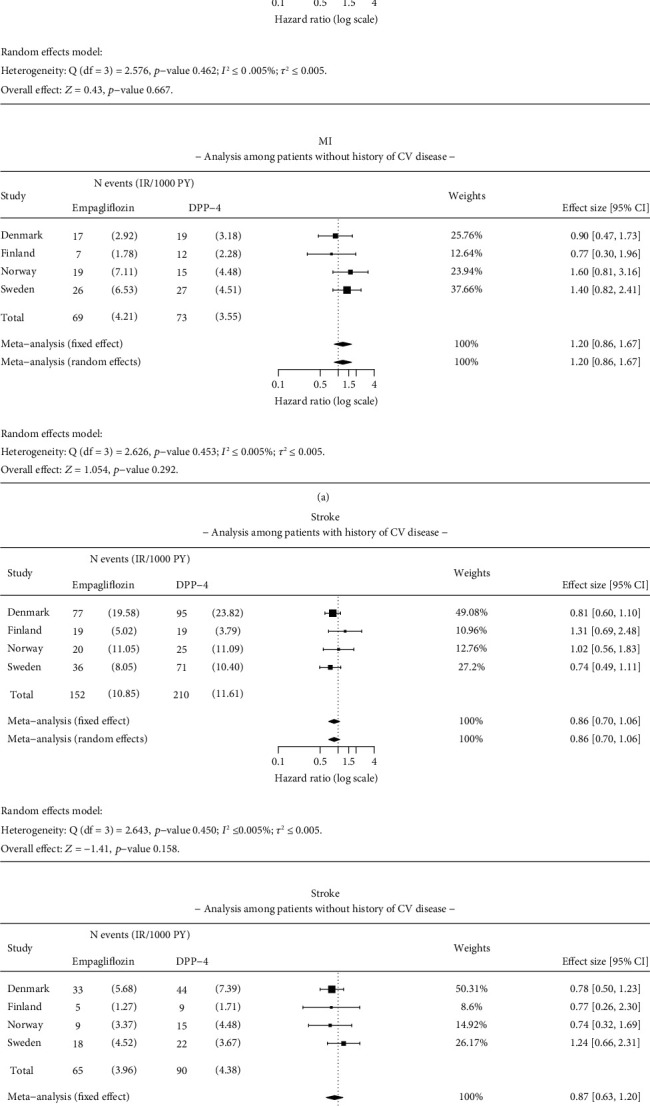
Results of the meta-analysis for (a) myocardial infarction (MI) and (b) stroke among patients with and without pre-existing cardiovascular (CV) disease. Analysis details: As-treated (AT). Numbers < 5 are not shown due to data protection, but they are included in meta-analysis. If values < 5 exist, a total number of events and incidence rates are presented as intervals. The time window for CV disease is ever before the index date.

Results were consistent with the main analysis for ACM, HHF, MI, and stroke in patients with and without a history of CV disease at baseline (Figures 7 and 8). The HR for empagliflozin versus DPP-4i for ACM was 0.50 (95% CI 0.41–0.61) in patients with a history of CV disease and 0.54 (95% CI 0.42–0.69) in patients without a history of CV disease, at baseline (Figure 7(a)). Significant reduction in risk of HHF was found in patients with CV disease at baseline (HR 0.68; 95% CI 0.49–0.94) and without CV disease (HR 0.42; 95% CI 0.21–0.85) between empagliflozin and DPP-4i initiators (Figure 7(b)). No significant difference was observed in the risk of MI and stroke in patients with or without a history of CV disease at baseline (Figure 8).

### 3.5. Sensitivity Analyses

**Figure 9:**
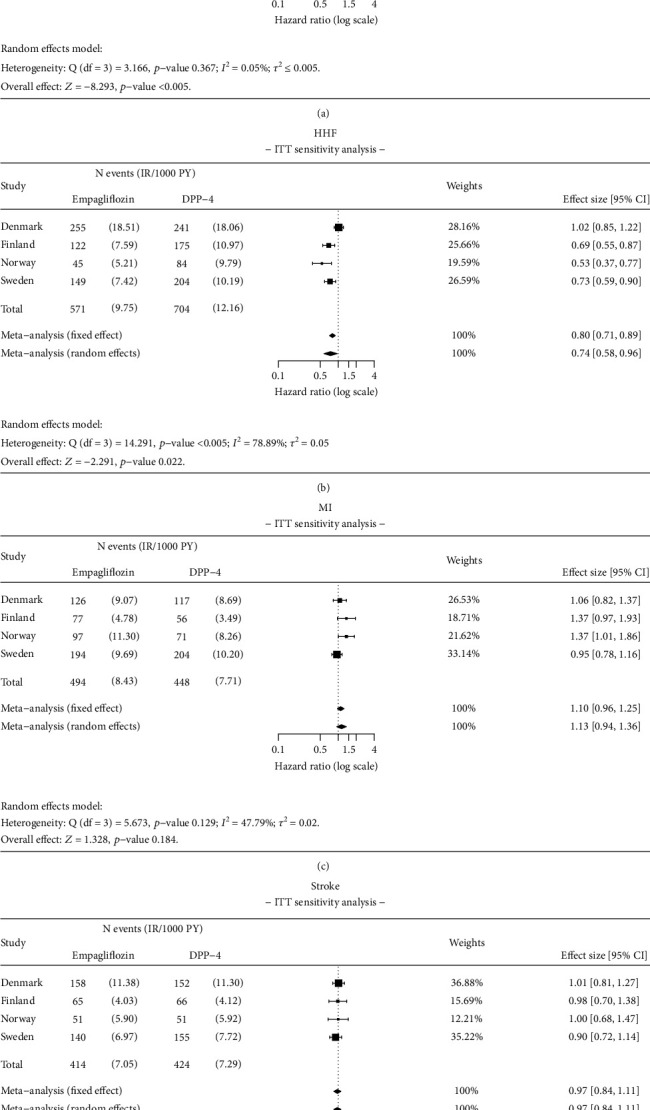
Results of ITT sensitivity analysis for (a) all-cause mortality (ACM), (b) hospitalization for heart failure (HHF), (c) myocardial infarction (MI), and (d) stroke. Analysis details: Intention-to-treat (ITT). Numbers < 5 are not shown due to data protection, but they are included in meta-analysis. If values < 5 exist, total number of events and incidence rates are presented as intervals.

Using an ITT approach, results were consistent with the main AT analysis. Initiation of empagliflozin was associated with 31% significantly lower risk (HR 0.69; 95% CI 0.63–0.75) for ACM and 26% significantly lower risk (HR 0.74; 95% CI 0.58–0.96) for HHF when compared to DPP-4i (Figure 9).

## 4. Discussion

This study demonstrates the beneficial effects associated with initiation of empagliflozin as compared to DPP-4i (i.e., significantly lower risk for ACM, HHF, CVM, and ESRD outcomes) are also observed in Nordic patient populations when examined in real-world clinical practice settings. These observations were similar between patients both with and without baseline CV disease. These findings fill evidence gaps regarding the effectiveness of the specific SGLT2i empagliflozin in clinical practice. The findings of this investigation are consistent with several previous studies, including the EMPRISE (Empagliflozin Comparative Effectiveness and Safety) US study, which reported that empagliflozin was associated with 49% significantly lower risk for HHF compared to sitagliptin (HR 0.51; 95% CI, 0.39–0.68) and 44% significantly lower risk compared to any DPP-4i (HR 0.56; 95% CI, 0.43–0.73). Results from the EMPRISE (Empagliflozin Comparative Effectiveness and Safety) US study also reported similar risk for MI or stroke between empagliflozin and DPP4i, which are findings similar to those reported in the current EMPRISE (Empagliflozin Comparative Effectiveness and Safety) study [14]. Results from the East Asian regional subgroup EMPRISE (Empagliflozin Comparative Effectiveness and Safety) study were also similar to those from this EMPRISE (Empagliflozin Comparative Effectiveness and Safety) Nordic study in that significantly lower risk for HHF (HR 0.82; 95% CI 0.71–0.94) and ACM (HR 0.64; 95% CI 0.50–0.81) observed in the empagliflozin group compared to DPP4is [23]. The meta-analyses of patients with T2D from South Korea, Japan, Singapore, Israel, Australia, and Canada (the CVD-REAL-2 study) also reported significantly lower risks for ACM (HR 0.51; 95% CI 0.37–0.70), HHF (HR 0.64; 95% CI 0.50–0.82) in SGLT2i initiators versus other glucose-lowering medications [16]. The CVD-REAL Nordic study, which included data from Denmark, Norway, and Sweden, also reported that SGLT2i were associated with significantly decreased risk of HHF (HR 0.70; 95% CI 0.61–0.81) and CVM (HR 0.53; 95% CI 0.40–0.71) compared with other glucose-lowering medications in the general T2D cohort. Further, the CVD-REAL Nordic study found no difference in the overall risk of either stroke or MI outcomes between patients using SGLT2i and patients using other glucose-lowering medications [18]. Overall, there is limited information available on the risks for MI and stroke among patients with T2D using empagliflozin compared to placebo or other glucose-lowering treatments due to similar limitations in existing studies. Therefore, the results of this study provided additional insights on the effectiveness of empagliflozin on MI and stroke among T2D patients in routine clinical care settings.

The findings of this study build upon the findings of the EMPA-REG OUTCOME trial for patients with T2D and CV disease receiving standard conventional therapy [8]. We observed, in routine clinical practice settings, results similar to those reported in the EMPA-REG OUTCOME trial. The significantly lower risk for HHF and CVM was similar to that observed in the EMPA-REG OUTCOME trial where a 35% and 38% relative risk reduction was observed with empagliflozin use versus placebo for HHF and CVM, respectively. Furthermore, in the current study, a significantly lower risk of ESRD was observed with empagliflozin compared to DPP-4i, although these results must be interpreted cautiously given the relatively low number of observed events and limited follow-up time. These EMPRISE (Empagliflozin Comparative Effectiveness and Safety) Nordic findings further support the overall slower progression of kidney disease and lower rates of renal events observed in the EMPA-REG OUTCOME trial [9] and in the EMPA-KIDNEY trial [10].

In contrast to previous observational studies [12, 13, 17], this EMPRISE (Empagliflozin Comparative Effectiveness and Safety) study is aimed at improving the balance between treatment groups and reducing the likelihood of confounding and time-related biases by applying PS matching and using an active comparator, incident (new) user study design. Furthermore, these results are reflective of outcomes examined in routine clinical care settings in Nordic countries, and they include active comparators that represent appropriate treatment alternatives to empagliflozin. The data included in this Nordic study were taken from comprehensive nationwide registers, which nearly cover entire national populations [24]. The PS methodology adjusted for ≥ 105 covariates, including baseline insulin and diabetes medication use and common comorbidities associated with diabetes and healthcare utilization, which may all be considered proxies for potential confounders, such as diabetes severity and duration that were not included in the registers (except for Sweden). Additionally, T2D-related laboratory information was controlled for in the Danish and Swedish analyses, which accounted for additional potential residual confounding (e.g., glycated hemoglobin (HbA1c) and estimated glomerular filtration rate (eGFR)).

Since this noninterventional, multicountry cohort study used secondary data, the availability and coverage of the study outcomes varied across the study countries. Further, as this is an observational study, it is unlikely that all residual confounding factors were fully accounted for (e.g., incomplete recording of diagnoses and potential lack of variables in the data sources). As nonadherence in chronic therapy is recognized to be a problem in routine clinical care, the main analyses were carried out using an AT approach. Since this analytic approach accounts for patterns of nonadherence, it enhances comparability between national analyses. To consider biases associated with informative censoring and exposure misclassification in the AT approach, ITT analyses were conducted as sensitivity analyses. Risk reductions for all end points were lower in the sensitivity analyses using an ITT approach compared to the AT approach, yet risk differences remained statistically significant. However, since actual drug use could not be confirmed using Nordic register-based data (since it relied solely on filled prescriptions), some exposure misclassification could still influence the ITT analyses. To fully account for the causal effect associated with time-varying glucose-lowering treatment and potential time-varying covariates such as serum lipid profile or eGFR, additional models such as the marginal structural model may be needed [25]. There is a limited possibility that patients may have received DPP-4i prior to the washout period. However, a 1-year washout period should be sufficient in clinical settings as patients typically quickly switch between glucose-lowering agents or add treatments to the baseline therapy. Further, some DPP-4i, particularly saxagliptin, alogliptin, and linagliptin, may be associated with different risks of HHF [26–28].

Due to the low numbers of renal events in Norway and Sweden, it is important to interpret the ESRD findings with caution. The differences seen in ESRD events across the Nordic countries may be due to multiple reasons, such as differences in implementation of guidelines for glucose-lowering treatment in case of renal failure, or different hospital reimbursements associated with the diagnostic codes. Additionally, these differences may also be due to outcome misclassification or because of confounding by baseline level of eGFR due to prescribing restrictions in low eGFR. Further, heterogeneity was seen in the meta-analyses relating to ACM, ESRD, and HHF risk. This affects the generalizability of the effect estimates. Despite the limitations, this study reflects the differences in risk observed across treatments in the broad population of patients encountered in regular clinical settings across the various healthcare systems in four Nordic countries. Although the average follow-up time in this study was sufficient due to the large study population, there were analyses where the follow-up time was generally less than 1 year. This may limit the detection of differences in outcomes that may occur later during empagliflozin or DPP-4i treatment.

## 5. Conclusions

In conclusion, initiation of empagliflozin was associated with a significantly lower risk of ACM, HHF, CVM, and ESRD compared to DPP-4i in patients with T2D undergoing routine glucose-lowering therapy in Nordic countries. The results were consistent in patients with or without CV disease and CHF at the time of treatment initiation, after accounting for a large number of potential clinical confounders. The findings are by large considered generalizable to other real-world populations as Nordic data sources used in this study are nationwide, and therefore, selection bias is minimal.

## Data Availability

The patient-level data that support the findings of this study is not available from third-party data vendors, due to public Nordic data legislation.

## Supporting Information

Supporting Information Additional supporting information can be found online in the Supporting Information section. The supporting information provides additional details regarding (A) the characteristics of data sources from the four Nordic countries; (B) the definitions of exposure periods; (C) definitions of covariates, propensity score variables, and laboratory values; (D) definitions for the study outcomes; and (E) the baseline patient characteristics by country and study subgroup. Description of the data sources in four Nordic countries. This study is based on several nationwide data sources of observational data (national registers) in four Nordic countries, namely, Denmark, Finland, Norway, and Sweden. Three types of national registers were used in this study for all Nordic countries: patient registers, prescription registers, and cause of death registers. Additionally, national, or regional registers containing laboratory values and lifestyle factors were utilized. Patients with dispensations of empagliflozin, or any dipeptidyl peptidase-4 inhibitor (DPP-4i), were identified in the prescription registers. The identified population was then linked to the other registers used in this study. All data was deidentified, and unique individual patient identification numbers were available for all data sources which allowed for extensive linkage between data sets in each country. For Finland, data on socioeconomic status was also extracted. Due to Norwegian regulations and the pseudonymization of the prescription register, identification of patients was a two-step process: first by diagnosis (at any position) in inpatient, outpatient, or primary care and then by adding prescription data to identified subjects. In this country, International Classification of Primary Care, 2^nd^ edition (ICPC-2) codes were used to identify type 2 diabetes (T2D) patients in primary care (the “Kontroll og utbetaling av helserefusjoner” (KUHR) register) and the International Classification of Diseases and Related Health Problems (ICD-10) codes in inpatient/outpatient (the national patient register). The utilized data sources are specified in Table S1. Table S1. Summary of data sources per Nordic country (Denmark, Finland, Norway, Sweden). Definition of exposure periods. Exposure periods were defined based on available data on drug dispensations or other records of the drug use in each country. Drug use was assumed to begin on the date of a dispensation (Figure S1). A supply indicating the duration of exposure after a dispensation was defined for each dispensation based on the amount purchased (i.e., number of pills and strength per pill). Hence, the duration of the supply in days (exposure period) was derived from the total dispensed amount divided by dose per day: Number of packages^∗^package size^∗^strength (total mg)/[(daily defined dose, DDD (mg)] = drug use in DDDs. The calculated drug use in daily defined doses (DDDs) corresponded to the number of day's supply per prescription. The DDDs were based on the World Health Organization (WHO) DDD, unless a prescribed dose for the specific purchase was recorded or a national DDD was defined. If a subsequent supply started before the previous supply had finished, the start of the subsequent supply was shifted. For example, if a patient received the first supply for 60 days and refilled at day 50 for another 60 days, the duration of the two supplies combined were 50 + 10 + 60 = 120 days, after shifting the start of the second supply with 10 days for which the patient had supply in storage from the first supply. To avoid artificially long exposure periods, however, a subsequent supply was shifted with a maximum of 14 days, which was considered a reasonable time for patients to refill prior to the end of their ongoing supply. Further, a subsequent supply could not be shifted over the grace period (as defined below). Figure S1. Illustration of combining exposure periods with grace periods and the differences between the AT and ITT approaches. AT = as-treated; GP = grace period; ITT = intention-to-treat. A grace period of 100% of the duration of the most recent supply was included in the exposure period to account for uncertainty related to actual drug use patterns. The grace period was defined from the most recent supply. In the above example on two overlapping supplies with 60 days, totaling 120 days, the grace period was 60 days (100% of the most recent supply). Overlapping supplies and grace periods were combined as exposure periods (Figure S1). The discontinuation was defined as the date of ending the grace period (Figure S2). The switch of a drug was defined as replacing one study drug with another, and no grace period was used (Figure S2). The concomitant use was defined as the simultaneous use of study drugs, and the first date of concomitant use ended the follow-up, that is, no grace period was used (Figure S2). Figure S2. Illustration of a study drug (a) discontinuation, (b) switch, and (c) concomitant use. An “as-treated” (AT) approach was utilized in the main analyses. This means that the follow-up is censored at discontinuation, switch to another study drug, or concomitant use, as detailed in Figure S1. Sensitivity analyses for the primary effectiveness outcomes were performed utilizing an ‘intention-to-treat' (ITT) approach. In the ITT analysis, the exposure was assumed to continue until the occurrence of effectiveness outcome, death, or end of data availability. However, changes in drug treatment, that is, discontinuation, switch, or concomitant use, did not end the follow-up (details provided in Figure S1 and Figure S2). Therefore, the follow-up in the ITT analyses is at least as long or longer than in the AT analyses. The ITT approach was accounted for the possibility that outcomes associated with the drug exposure might emerge after discontinuation. Table S2. Definitions of covariates and propensity score variables. Table S3. Definitions of laboratory values. Table S4. Definitions of study outcomes. Table S5. Baseline characteristics for empagliflozin and DPP-4i subcohorts after PS-matching.
