# Supplementary material for: Empagliflozin Use Is Associated With Lower Risk of All-Cause Mortality, Hospitalization for Heart Failure, and End-Stage Renal Disease Compared to DPP-4i in Nordic Type 2 Diabetes Patients: Results From the EMPRISE (Empagliflozin Comparative Effectiveness and Safety) Study
Source: J Diabetes Res. 2024 Oct 12;2024:6142211. doi: 10.1155/2024/6142211 (PMC11490347; doi:10.1155/2024/6142211)
Supplement: Supporting Information — Additional supporting information can be found online in the Supporting Information section. The supporting information provides additional details regarding (A) the characteristics of data sources from the four Nordic countries; (B) the definitions of exposure periods; (C) definitions of covariates, propensity score variables, and laboratory values; (D) definitions for the study outcomes; and (E) the baseline patient characteristics by country and study subgroup. Description of the data sources in four Nordic countries. This study is based on several nationwide data sources of observational data (national registers) in four Nordic countries, namely, Denmark, Finland, Norway, and Sweden. Three types of national registers were used in this study for all Nordic countries: patient registers, prescription registers, and cause of death registers. Additionally, national, or regional registers containing laboratory values and lifestyle factors were utilized. Patients with dispensations of empagliflozin, or any dipeptidyl peptidase-4 inhibitor (DPP-4i), were identified in the prescription registers. The identified population was then linked to the other registers used in this study. All data was deidentified, and unique individual patient identification numbers were available for all data sources which allowed for extensive linkage between data sets in each country. For Finland, data on socioeconomic status was also extracted. Due to Norwegian regulations and the pseudonymization of the prescription register, identification of patients was a two-step process: first by diagnosis (at any position) in inpatient, outpatient, or primary care and then by adding prescription data to identified subjects. In this country, International Classification of Primary Care, 2nd edition (ICPC-2) codes were used to identify type 2 diabetes (T2D) patients in primary care (the “Kontroll og utbetaling av helserefusjoner” (KUHR) register) and the International Classification of Diseases and Rela [file 6142211.f1.zip › Supplementary Table 1.docx]

Table 1. Summary of data sources per Nordic country (Denmark, Finland, Norway, Sweden).

| **Data source** | **Data category** | **Relevant information** | **Coverage** | **Updates** |
| --- | --- | --- | --- | --- |
| **Denmark** | | | | |
| Danish National Patient Register | Inpatient and specialized outpatient care | Dates of visits, primary and secondary diagnoses (ICD-10), procedure codes, etc. | National | Annually |
| Registry of Medicinal Product Statistics | Filled Prescriptions* | Date of prescription and dispatch, ATC-code, strength, administration form, drug name, cost of prescription | All dispensing from Danish pharmacies | Semi-annual |
| Danish Causes of Death Registry | Mortality | Date of death and cause | National | Annually |
| The clinical laboratory information system (LABKA) | Laboratory data | Clinical laboratory information | Regional | Annually |
| Danish Adult Diabetes Registry (DVDD) | Hospital and general practice | Quality of treatment and follow-up | National | Annually |
| Danish Registry of Diabetic Retinopathy (DiaBase) | Hospital eye departments and private ophthalmologic al practice | Diabetic eye disease and screening programs | National | Annually |
| Public Health Insurance Register (NHIR) | Public health insurance system | Recipients, services and related costs | National | Annually |
| **Finland** | | | | |
| Care Register for Health Care (HILMO) | Inpatient and specialized outpatient care | Dates of visits, primary and secondary diagnoses (ICD-10), procedure codes, etc. | National | Annually |
| Tilastokeskus (Statistics Finland) | Socioeconomic variables | Socioeconomic status | National | Annually |
| Finnish Prescription Registry of reimbursed drugs | Filled Prescriptions* | Date of prescription, date of dispatch, ATC-code, strength, administration form, name of drug, and cost of prescription | National | Monthly |
| Finnish Causes of Death Registry | Mortality | Date of death and cause | All deaths in Finland | Annually |
| Fimlab | Laboratory data | Clinical laboratory information | Regional |  |
| HUSLAB | Laboratory data | Clinical laboratory information | Regional |  |
| TYKSlab | Laboratory data | Clinical laboratory information | Regional |  |
| **Norway** | | | | |
| Norwegian Patient Register | Inpatient and specialized outpatient care | Dates of visits, primary and secondary diagnoses (ICD-10), procedure codes, etc. | National | Quarterly |
| Norwegian Prescription Database | Filled Prescriptions* | Date of prescription, date of dispatch, ATC-code, strength, administration form, name of drug, and cost of prescription | National (All dispensing from Norwegian pharmacies) | Monthly |
| KUHR | Primary care | Dates of visits, primary and secondary diagnoses (ICPC2) | National |  |
| Norwegian Causes of Death Registry | Mortality | Date of death and cause | National | Annually |
| **Sweden** | | | | |
| National patient register | Inpatient and specialized outpatient care (physician visits only) | Dates of visits, primary and secondary diagnoses (ICD-10), procedure codes, etc. | National | Annually |
| The national dispensed drug register | Prescriptions* | Date of prescription, date of dispatch, ATC-code, strength, administration form, name of drug, and cost of prescription | National (All dispensing from Swedish pharmacies) | Monthly |
| The cause of death register | Mortality | Date of death and cause | All deaths in Sweden | Annually |
| The Swedish National Diabetes Register (NDR) | Laboratory data, Lifestyle factors | Clinical laboratory information, smoking and BMI | National (~80% of diabetes patients) | Annually |
| * Over-the-counter drug data not included/available  ATC=Anatomical Therapeutic Chemical code; BMI=body mass index; ICD-10=the International Classification of Diseases and Related Health Problems, 10^th^ revision | | | | |
