# Supplementary material for: Empagliflozin Use Is Associated With Lower Risk of All-Cause Mortality, Hospitalization for Heart Failure, and End-Stage Renal Disease Compared to DPP-4i in Nordic Type 2 Diabetes Patients: Results From the EMPRISE (Empagliflozin Comparative Effectiveness and Safety) Study
Source: J Diabetes Res. 2024 Oct 12;2024:6142211. doi: 10.1155/2024/6142211 (PMC11490347; doi:10.1155/2024/6142211)
Supplement: Supporting Information — Additional supporting information can be found online in the Supporting Information section. The supporting information provides additional details regarding (A) the characteristics of data sources from the four Nordic countries; (B) the definitions of exposure periods; (C) definitions of covariates, propensity score variables, and laboratory values; (D) definitions for the study outcomes; and (E) the baseline patient characteristics by country and study subgroup. Description of the data sources in four Nordic countries. This study is based on several nationwide data sources of observational data (national registers) in four Nordic countries, namely, Denmark, Finland, Norway, and Sweden. Three types of national registers were used in this study for all Nordic countries: patient registers, prescription registers, and cause of death registers. Additionally, national, or regional registers containing laboratory values and lifestyle factors were utilized. Patients with dispensations of empagliflozin, or any dipeptidyl peptidase-4 inhibitor (DPP-4i), were identified in the prescription registers. The identified population was then linked to the other registers used in this study. All data was deidentified, and unique individual patient identification numbers were available for all data sources which allowed for extensive linkage between data sets in each country. For Finland, data on socioeconomic status was also extracted. Due to Norwegian regulations and the pseudonymization of the prescription register, identification of patients was a two-step process: first by diagnosis (at any position) in inpatient, outpatient, or primary care and then by adding prescription data to identified subjects. In this country, International Classification of Primary Care, 2nd edition (ICPC-2) codes were used to identify type 2 diabetes (T2D) patients in primary care (the “Kontroll og utbetaling av helserefusjoner” (KUHR) register) and the International Classification of Diseases and Rela [file 6142211.f1.zip › Supplementary Table 2.docx]

Table 2. Definitions of covariates and propensity score variables

| **Covariate** | **Definition** | **Information used in identifying the outcome** | | | |
| --- | --- | --- | --- | --- | --- |
|  |  | **ICD-10 codes** | **NCSP/non-surgical procedure codes (any position)** | **Laboratory measurements** | **ATC codes** |
| **Demographics and Lifestyle** | | | | | |
| Age^1-4^ | Age in years at index date | No | No | No | No |
| Sex^1-4^ | Female, male | No | No | No | No |
| Socioeconomic status^2^ | Latest (on/before index) registered occupation by category: (10) Farmer etc. entrepreneurs, (20) Other entrepreneurs, (31) Senior officials and upper management, (32) Senior officials and employees in research and planning, (33) Senior officials and employees in education and training, (34) Other senior officials and employees, (41) Supervisors, (42) Clerical and sales workers, independent work, (43) Clerical and sales workers, routine work, (44) Other lower-level employees, (51) Workers in agriculture, forestry and commercial fishing, (52) Manufacturing workers, (53) Other production workers, (54) Distribution and service workers, (60) Students, (70) Pensioners, (81) Unemployed, (82) Conscripts, persons in non-military service, (99) Unknown. Finland only | No | No | No | No |
| Calendar time by 3-month block^1-4^ | Defined as the block by year that the index date corresponds to:  1 Jan - 31 Mar, 1 Apr - 30 Jun, 1 Jul - 30 Sep, or 1 Oct - 31 Dec | No | No | No | No |
| Obesity^1-4^ | ≥1 recorded diagnosis at any time in the past (maximum look-back January 2005; except for Swedish prescription data [July 2005] and for Norwegian patient data [January 2008]) related to obesity OR BMI of ≥30 (Sweden only, through data from the national diabetes registry) OR  drug filled dispensations related to obesity care | E66 | No | No | A08A |
| Overweight^4^ | Defined as BMI of ≥25 and <30 (Sweden only, through data from the national diabetes registry) indicating overweight at any time in the past | No | No | No | No |
| Smoking^1-4^ | ≥1 recorded diagnosis or status (Sweden only, through data from the national diabetes registry) indicating smoking at any time in the past (maximum look-back January 2005; except for Norwegian patient data [January 2008]) | F17, Z72.0 | No | No | No |
| Alcohol abuse or dependence^1-4^ | ≥1 recorded diagnosis indicating alcohol abuse or dependence at any time in the past (maximum look-back January 2005; except for Norwegian patient data [January 2008]) | F10, K70, G62.1, I42.6, Z71.4 | No | No | No |
| Drug abuse or dependence^1-4^ | ≥1 recorded diagnosis indicating alcohol drug or dependence at any time in the past (maximum look-back January 2005; except for Norwegian patient data [January 2008]) | F11, F12, F13, F14, F15, F16, F18, F19, Z71.5 | No | No | No |
| **Diabetes History and Complications** | | | | | |
| Time since T2D diagnosis^2-4^ | Whichever is the longest time since: Time in years since first recorded diagnosis of T2D  OR  Time in years since first dispensed prescription of metformin (including fixed-dose combinations with other antidiabetic drugs) OR as indicated by laboratory data sources (Sweden and Denmark only) | E11 | No | Sweden (NDR): variable "debutalder" Denmark (DVDD): variable "Diagnosetidspunkt" | A10BA02, A10BD15, A10BD20, A10BD16 (only in Sweden), A10BD23, A10BD07, A10BD08, A10BD10, A10BD13 (not in NOR), A10BD11, A10BD05, A10BD03 (only in Denmark) |
| Number of prior antidiabetic drug classes^2-4^ | Total number of past different antidiabetic drug classes with ≥1 filled dispensation at any time in the past (maximum look-back January 2005; except for Swedish prescription data [July 2005]). Classes are grouped ATC codes and defined by each bracket in the ATC column | No | No | No | A10BA02, A10BD17, A10BD05, A10BD14, A10BD03, A10BD02, A10BB12, A10BB07, A10BB04, A10BG, A10BD06, A10BH01, A10BH02, A10BH03, A10BH04, A10BH05, A10BK01, A10BK02, A10BK03, A10BK04, A10BJ, A10BX02, A10BX03, A10BX08, A10BF, A10BD17, A10BD07, A10BD08, A10BD10, A10BD11, A10BD13, A10BD15, A10BD16, A10BD20, A10BD23 |
| Diabetic retinopathy^1-4^ | ≥1 recorded diagnosis of diabetic retinopathy at any time in the past (maximum look-back January 2005; except for Norwegian patient data [January 2008]) | H36.0, E10.3, E11.3, E13.3 (excluding codes ending with 36, 37, 39; Exx.36, Exx.37, Exx.39) | No | No | No |
| Diabetes with other ophthalmic manifestations^2-4^ | ≥1 recorded diagnosis at any time in the past (maximum look-back January 2005; except for Norwegian patient data [January 2008]) related to  diabetes with neurological manifestation (without mention of other retinal disorders),  OR diabetic cataract,  OR diabetic glaucoma | H28, H42, E10.3F (Sweden only) E10.3 + H28.0 (not Sweden), E10.3W (Sweden only), E11.3F (Sweden only), E11.3 + H28.0 (not Sweden), E10.3W (Sweden only), E13.3 + H28.0 (not Sweden) | No | No | No |
| Retinal detachment, vitreous hemorrhage, vitrectomy^1-4^ | ≥1 recorded diagnosis or procedure related to retinal detachment, vitreous hemorrhage, vitrectomy at any time in the past (maximum look-back January 2005; except for Norwegian patient data [January 2008]) | H33 (except H33.3), H43.1, H45.0 | CKD60, CKD65 | No | No |
| Retinal laser coagulation therapy^1-4^ | ≥1 recorded procedure related to retinal laser coagulation therapy at any time in the past (maximum look-back January 2005; except for Norwegian patient data [January 2008]) | No | Sweden/Norway/Finland: CKD40, Denmark: KCKD40 | No | No |
| Diabetic neuropathy^1-4^ | ≥1 recorded diagnosis related to diabetic neuropathy at any time in the past (maximum look-back January 2005; except for Norwegian patient data [January 2008]) | G99.0, G59.0, G63.2, E10.4, E11.4, E13.4 | No | No | No |
| Diabetic nephropathy^1-4^ | ≥1 recorded diagnosis related to diabetic nephropathy at any time in the past (maximum look-back January 2005; except for Norwegian patient data [January 2008]) | E10.2, E11.2, E13.2, N08 | No | No | No |
| Hypoglycaemia^2-4^ | ≥1 recorded diagnosis related to hypoglycemia  OR diabetes with other specified manifestations at any time in the past (maximum look-back January 2005; except for Norwegian patient data [January 2008]) | E16.0, E16.1, E16.2, T38.3 (only available in Sweden and Denmark), E10.6, E11.6, E10.6, E11.6 (except E1x.610 and E1x.641) | No | No | No |
| Hyperglycaemia^1-4^ | ≥1 recorded diagnosis related to hyperglycemia at any time in the past (maximum look-back January 2005; except for Norwegian patient data [January 2008]) | R73.9 | No | No | No |
| Disorders of fluid electrolyte and acid-base balance^1-4^ | ≥1 recorded diagnosis related to disorders of fluid electrolyte and acid-base balance at any time in the past (maximum look-back January 2005; except for Norwegian patient data [January 2008]) | E87, E86 | No | No | No |
| Diabetic keto/lactateacidosis^2-4^ | ≥1 recorded diagnosis related to diabetic keto/lactate acidosis at any time in the past (maximum look-back January 2005; except Norwegian patient data [January 2008]) | E111, E110 | No | No | No |
| Hyperosmolar hyperglycemic nonketotic syndrome (HONK)^2-4^ | ≥1 recorded diagnosis related to HONK at any time in the past (maximum look-back January 2005; except Norwegian patient data [January 2008]) | E11.0, E13.0 | No | No | No |
| Diabetes with peripheral circulatory disorders^1-4^ | ≥1 recorded diagnosis related to diabetes with peripheral circulatory disorders at any time in the past (maximum look-back January 2005; except Norwegian patient data [January 2008]) | E10.5, E11.5, E13.5, I79.8 | No | No | No |
| Diabetic foot^1,2,4^ | ≥1 recorded diagnosis related to diabetic foot at any time in the past (maximum look-back January 2005; except Norwegian patient data [January 2008]) | E10.6D (only Sweden), E11.6D (only Sweden), E10.5B (only Denmark), E11.5B (only Denmark) | No | No | No |
| Gangrene^1-4^ | ≥1 recorded diagnosis related to gangrene at any time in the past (maximum look-back January 2005; except Norwegian patient data [January 2008]) | R02, E10.5B (only Sweden), E11.5B (only Sweden), E10.5 (only Norway), E11.5 (only Norway), E13.5 (only Norway), E10.5A + E10.5C (only Denmark), E11.5A + E11.5C (only Denmark), E13.5A + E13.5C (only Denmark), E10.5 + I79.2 (only Finland), E11.5 + I79.2 (only Finland), E13.5 + I79.2 (only Finland) | No | No | No |
| Lower-extremity amputation^1-4^ | ≥1 recorded diagnosis related to lower limb amputation, with differentiation of major and minor amputation at any time in the past (maximum look-back January 2005; except Norwegian patient data [January 2008]) | Z89.4, Z89.5, Z89.7 | No | No | No |
| Osteomyelitis^1-4^ | ≥1 recorded diagnosis related to osteomyelitis at any time in the past (maximum look-back January 2005; except for Norwegian patient data [January 2008]) | M86 | No | No | No |
| Skin infections^1-4^ | ≥1 recorded diagnosis related to skin infections at any time in the past (maximum look-back January 2005; except Norwegian patient data [January 2008]) | L01, L02, L03, L04, L05, L08, L88, E83.2, K12.2, L92.8, B78.1 | No | No | No |
| Erectile dysfunction^1-4^ | ≥1 recorded diagnosis related to erectile dysfunction at any time in the past (maximum look-back January 2005; except Norwegian patient data [January 2008]) | N48.4, F52.2 | No | No | No |
| Diabetes with unspecified complication^1-4^ | ≥1 recorded diagnosis related to diabetes with unspecified complication at any time in the past (maximum look-back January 2005; except Norwegian patient data [January 2008]) | E10.8, E11.8, E13.8 | No | No | No |
| Diabetes mellitus without mention of complications^1-4^ | ≥1 recorded diagnosis related to diabetes mellitus without mention of complications at any time in the past (maximum look-back January 2005; except Norwegian patient data [January 2008]) | E10.9, E11.9, E13.9 | No | No | No |
| Hypertension^1-4^ | ≥1 inpatient OR ≥2 outpatient encounters with diagnosis of hypertension  OR   ≥1 dispensing of hypertension drugs at any time in the past (maximum look-back January 2005; except for Swedish prescription data [July 2005] and for Norwegian patient data [January 2008]) | I10-I16 | No | No | C09A, C09B, C09C, C09D, C07, C08, C07FB (only in Sweden), C09BB, C09DB, C03A, C03C, C03D, C01DA02, C01DA08 (not available in Sweden), C01DA14, C01EB18 (not available in Sweden and Norway), C02CA04 (not available in Finland), C03DA04, C02CA01 (only in Finland), G04CA03 (not available in Finland), C02AC01 (not available in Norway and Denmark), C02AC02, C02DB02 (not available in Finland and Denmark), C09XA02, C09XA52 (not available in Sweden) |
| Hyperlipidaemia^1-4^ | Any dispensing/prescription of any of the listed ATC codes for statins or other lipid-lowering drugs at any time in the past (maximum look-back January 2005; except for Swedish prescription data [July 2005] and for Norwegian patient data [January 2008]) | E78.0-E78.5 | No | No | C10 (except C10AA, C10BA, C10BX) |
| Ischemic heart disease^1-4^ | ≥1 recorded diagnosis of ischemic heart disease at any time in the past (maximum look-back January 2005; except Norwegian patient data [January 2008]) | I20-I25 | No | No | No |
| Acute MI^1-4^ | ≥1 recorded diagnosis of acute MI at any time in the past (maximum look-back January 2005; except Norwegian patient data [January 2008]) | I21-I22 | No | No | No |
| Acute coronary syndrome/unstable angina^2-4^ | ≥1 recorded diagnosis of acute coronary syndrome/unstable angina at any time in the past (maximum look-back January 2005; except Norwegian patient data [January 2008]) | I20-I22, I20.0 | No | No | No |
| Old MI^1-4^ | ≥1 recorded diagnosis of old MI and/or history of MI at any time in the past (maximum look-back January 2005; except Norwegian patient data [January 2008]) | I25.2, I21 | No | No | No |
| Stable angina^1-4^ | ≥1 recorded diagnosis of stable angina at any time in the past (maximum look-back January 2005; except Norwegian patient data [January 2008]) | I20.1, I20.8, I20.9 | No | No | No |
| Coronary atherosclerosis and other forms of chronic ischemic heart disease^2-4^ | ≥1 recorded diagnosis of coronary atherosclerosis and other forms of chronic ischemic heart disease at any time in the past (maximum look-back January 2005; except Norwegian patient data [January 2008]) | I25 (except I25.2) | No | No | No |
| Coronary revascularization procedure^2-4^ | ≥1 recorded procedure for coronary atherosclerosis and other forms of chronic ischemic heart disease at any time in the past (maximum look-back January 2005; except Norwegian patient data [January 2008]) | No | FNA00, FNA10, FNA20, FNA96, FNB00, FNB20, FNB96, FNC10, FNC20, FNC30, FNC40, FNC50, FNC60, FNC96, FND10, FND20, FND96, FNE00, FNE10, FNE20, FNE96, FNF00, FNF10, FNF20, FNF30, FNF96, FNG00, FNG02, FNG05, FNG06, FNG10, FNG30, FNG96 | No | No |
| Other atherosclerosis^1-4^ | ≥1 recorded diagnosis at any time in the past (maximum look-back January 2005; except Norwegian patient data [January 2008]) of arteriosclerotic cardiovascular disease, OR Generalized and unspecified atherosclerosis | I25.1, I70.9 | No | No | No |
| History of CABG or PTCA^1-4^ | ≥1 recorded history of CABG or PTCA at any time in the past (maximum look-back January 2005; except Norwegian patient data [January 2008]) | Z95.1, Z95.5 | No | No | No |
| Any stroke^1-4^ | ≥1 recorded diagnosis of stroke at any time in the past (maximum look-back January 2005; except Norwegian patient data [January 2008]) | I60, I61, I63, I64, I67.89 | No | No | No |
| Ischemic stroke (with and without mention of cerebral infarction)^2-4^ | ≥1 recorded diagnosis of ischemic stroke at any time in the past (maximum look-back January 2005; except Norwegian patient data [January 2008]) | I63, I64 | No | No | No |
| Hemorrhagic stroke^2-4^ | ≥1 recorded diagnosis of hemorrhagic stroke at any time in the past (maximum look-back January 2005; except Norwegian patient data [January 2008]) | I60, I61 | No | No | No |
| Transient cerebral ischemia^1-4^ | ≥1 recorded diagnosis of transient cerebral ischemia at any time in the past (maximum look-back January 2005; except Norwegian patient data [January 2008]) | G45, I67.8 | No | No | No |
| Other cerebrovascular disease^1-4^ | ≥1 recorded diagnosis of transient cerebral ischemia at any time in the past (maximum look-back January 2005; except Norwegian patient data [January 2008]) | G45.4, G46.3, G46.4, G46.5, G46.6, G46.7, G46.8, I62, I67, I68 (except I67.3) | No | No | No |
| Late effects of cerebrovascular disease^1-4^ | ≥1 recorded diagnosis of late effects of cerebrovascular disease at any time in the past (maximum look-back January 2005; except Norwegian patient data [January 2008]) | I69 | No | No | No |
| Cerebrovascular procedure^2-4^ | ≥1 recorded procedures related to Carotid bypass,  OR Cerebrovascular revascularization at any time in the past (maximum look-back January 2005; except Norwegian patient data [January 2008]) | No | PAH20, PAH21, PAH25, PAB20-22, PAC20-22, PAE20-22, PAF20-22, PAG20-21, PAH20-21, PAK20-21, PAM21-22, PAN20-21, PAP20-21, PAQ20-21, PAR20-21 | No | No |
| Congestive heart failure (CHF) ^1-4^ | ≥1 recorded diagnosis of CHF at any time in the past (maximum look-back January 2005; except Norwegian patient data [January 2008]) | I11.x, I13.x, I50 | No | No | No |
| Peripheral vascular disease or surgery^1-4^ | ≥1 recorded diagnosis related to  peripheral vascular disease  OR ≥1 recorded procedure related to lower-extremity endarterectomy, stenting, angioplasty, or atherectomy, OR Lower-extremity bypass, OR Other peripheral vascular surgery at any time in the past (maximum look-back January 2005; except Norwegian patient data [January 2008]) | I70.2-I70.3, I73.9 | PEN, PEP, PEQ, PER, PES, PFN, PFP, PFQ, PFR, PFS, PEH, PFH, all PB, PE and PF | No | No |
| Atrial fibrillation (AF) ^1-4^ | ≥1 recorded diagnosis of AF at any time in the past (maximum look-back January 2005; except for Swedish prescription data [July 2005] and for Norwegian patient data [January 2008]) | I48 | No | No | No |
| Other cardiac dysrhythmia^1-4^ | ≥1 recorded diagnosis of other cardiac dysrhythmia at any time in the past (maximum look-back January 2005; except Norwegian patient data [January 2008]) | I47, I49 | No | No | No |
| Cardiac conduction disorders^1-4^ | ≥1 recorded diagnosis of cardiac conduction disorders at any time in the past (maximum look-back January 2005; except Norwegian patient data [January 2008]) | I44, I45 | No | No | No |
| Other CV disease^1-4^ | Any registered CV event at any time prior to index date (maximum look-back January 2005; except Norwegian patient data [January 2008]) | I00-I09, I30-I43, I71-I75, I77, I79, M30, M31 (except I72.2, I79.8, I73.9) | No | No | No |
| Peptic ulcer disease^1-4^ | ≥1 recorded diagnosis of peptic ulcer disease at any time in the past (maximum look-back January 2005; except Norwegian patient data [January 2008]) | K25–K28 | No | No | No |
| Edema^1-4^ | ≥1 recorded diagnosis of edema at any time in the past (maximum look-back January 2005; except Norwegian patient data [January 2008]) | R60 | No | No | No |
| Chronic pulmonary disease^1-4^ | ≥1 recorded diagnosis of chronic pulmonary disease at any time in the past (maximum look-back January 2005; except Norwegian patient data [January 2008]) | I27.8, I27.9, J40-J47, J60-J67, J68.4, J70.1, J70.3 | No | No | No |
| COPD^1-4^ | ≥1 recorded diagnosis of COPD at any time in the past (maximum look-back January 2005; except Norwegian patient data [January 2008]) | J44 | No | No | No |
| Asthma^1-4^ | ≥1 recorded diagnosis of asthma at any time in the past (maximum look-back January 2005; except Norwegian patient data [January 2008]) | J45 | No | No | No |
| Obstructive sleep apnea^1-4^ | ≥1 recorded diagnosis of obstructive sleep apnea at any time in the past (maximum look-back January 2005; except Norwegian patient data [January 2008]) | G47.3 (not Denmark), G47.32 (only Denmark) | No | No | No |
| Pneumonia^1-4^ | ≥1 recorded diagnosis of pneumonia at any time in the past (maximum look-back January 2005; except Norwegian patient data [January 2008]) | J10.0, J11.0, J12-J18, J69, A22.1, A48.1, B25.0, B44.0, A37.9 + J17.0 (only available in Finland), B77.8 + J17 (only available in Finland) | No | No | No |
| Renal dysfunction (non-diabetic)^2-4^ | ≥1 recorded diagnosis of renal dysfunction (non-diabetic) at any time in the past (maximum look-back January 2005; except Norwegian patient data [January 2008]) | See covariates acute renal disease, chronic renal disease, hypertensive nephropathy, miscellaneous renal insufficiency below | No | No | No |
| Acute renal disease^1^ | ≥1 recorded diagnosis of acute renal disease at any time in the past (maximum look-back January 2005; except Norwegian patient data [January 2008]) | K76.7, N00, N01, N08, N17, R82.1, R82.3 | No | No | No |
| Acute kidney injury that requires dialysis (ARF-D)^2-4^ | ≥1 recorded diagnosis of ARF-D AND  ≥1 procedure for hemodialysis and peritoneal dialysis  at any time in the past (maximum look-back January 2005; except Norwegian patient data [January 2008]) | N17 | Sweden: DR012, DR013, DR014, DR015, DR017, DR023; NOR: JAGD32, JAGD50, RXGD20, RXGD30; DEN: BJFD00, BJFD01, BJFD21, BJFD22, BJFD23, BJFD24, BJFD25, BJFD26, BJFD27, ZZ4340, ZZ4345, ZZ4350; FINLAND: TK800, TK810, TK820 | No | No |
| Chronic renal insufficiency^2-4^ | ≥1 recorded diagnosis of chronic renal insufficiency at any time in the past (maximum look-back January 2005; except Norwegian patient data [January 2008]) | N03, N05, N06, N07, N08, N14, N15, N16, N17.1, N17.2, N18, N19, N26 (only in Sweden), N27, N26.9, M32.1 (not in Finland), N16.4 (only in Finland), N08.5 (only in Finland), M35.0, N16.4 (only Norway), M35.0E (only Denmark), N16.4 (only Finland), I12 (not in Sweden) | No | No | No |
| Chronic kidney disease^1-4^ | ≥1 recorded diagnosis of chronic kidney disease at any time in the past (maximum look-back January 2005; except Norwegian patient data [January 2008]) | N18 | No | No | No |
| Chronic kidney disease stage 3-4^1,4^ | ≥1 recorded diagnosis of chronic kidney disease stage 3-4 at any time in the past (maximum look-back January 2005; except Norwegian patient data [January 2008]) | N18.3 (not available in Finland), N18.4 (not available in Finland) | No | No | No |
| Hypertensive nephropathy^1-4^ | ≥1 recorded diagnosis of hypertensive nephropathy at any time in the past (maximum look-back January 2005; except Norwegian patient data [January 2008]) | I12, I13 | No | No | No |
| Dialysis^2-4^ | ≥1 recorded diagnosis OR procedure for dialysis at any time in the past (maximum look-back January 2005; except Norwegian patient data [January 2008]) | Z49, Z99.2 | Sweden: DR012, DR013, DR014, DR015, DR016, DR017, DR023; Norway: JAGD30, JAGD31, JAGD32, JAGD50, RXGD05, RXGD20, RXGD25, RXGD30.  Denmark: BJFD00, BJFD01, BJFD02, BJFD20, BJFD21, BJFD22, BJFD23, BJFD24, BJFD25, BJFD26, BJFD27, ZZ4340, ZZ4341, ZZ4345, ZZ4346, ZZ4350.  Finland: TK800, TK810, TK820 | No | No |
| Miscellaneous renal insufficiency^1-4^ | ≥1 recorded diagnosis of miscellaneous renal insufficiency at any time in the past (maximum look-back January 2005; except Norwegian patient data [January 2008]) | I70.1, I72.2, I82.3, M10.3, N02.8, N04, N13.4, N13.5, N13.7, N13.8, N13.9, N28, N29, N39.2, Q60, Q63, S37.0xxA (not in Sweden) (except N28.85, N28.86) | No | No | No |
| Mild liver disease^1^ | ≥1 recorded diagnosis of mild liver disease at any time in the past (maximum look-back January 2005; except Norwegian patient data [January 2008]) | B18, K70.0-K70.3, K70.9, K71.3-K71.5, K71.7, K73, K74, K76.0, K76.2-K76.4, K76.8, K76.9, Z94.4 | No | No | No |
| Moderate or severe liver disease^1^ | ≥1 recorded diagnosis of moderate or severe liver disease at any time in the past (maximum look-back January 2005; except Norwegian patient data [January 2008]) | I85.0, I85.9, I86.4, I98.2, K70.4, K71.1, K72.1, K72.9, K76.5, K76.6, K76.7 | No | No | No |
| Osteoarthritis^1-4^ | ≥1 recorded diagnosis of osteoarthritis at any time in the past (maximum look-back January 2005; except Norwegian patient data [January 2008]) | M15-M19 | No | No | No |
| Other arthritis, arthropathies and musculoskeletal pain^1-4^ | ≥1 recorded diagnosis of other arthritis, arthropathies and musculoskeletal pain at any time in the past (maximum look-back January 2005; except Norwegian patient data [January 2008]) | M00-M08, M11-M14, M22-M25, M32-M36, M43.3, M43.4, M43.5, M60-M72, M75-M79, R26.2, R29.8 (except M04.1, R25.2 (not Denmark), R25.2C (only Denmark), M79.2) | No | No | No |
| Dorsopathies^1-4^ | ≥1 recorded diagnosis of dorsopathies at any time in the past (maximum look-back January 2005; except Norwegian patient data [January 2008]) | M43.2, M43.6, M45-M54, M62.830 | No | No | No |
| Bone fractures^2-4^ | ≥1 recorded diagnosis of bone fracture at any time in the past (maximum look-back January 2005; except Norwegian patient data [January 2008]) | S72.0, S72.1, S72.2, S32.1, S32.2, S32.3, S32.4, S32.5, S32.6, S32.7, S32.8, S52, S42.2, S42.3, S42.4 | No | No | No |
| Falls^2-4^ | ≥2 hospitalization with recorded diagnosis of accidental fall at any time in the past (maximum look-back January 2005; except Norwegian patient data [January 2008]) | W00-W19 | No | No | No |
| Osteoporosis^1-4^ | ≥1 recorded diagnosis of osteoporosis OR bone fracture at any time in the past (maximum look-back January 2005; except Norwegian patient data [January 2008]) | M80, S72.0, S72.1, S72.2, S32.1, S32.2, S32.3, S32.4, S32.5, S32.6, S32.7, S32.8, S52, S42.2, S42.3, S42.4 | No | No | No |
| Hyperthyroidism^1-4^ | ≥1 recorded diagnosis of hyperthyroidism at any time in the past (maximum look-back January 2005; except Norwegian patient data [January 2008]) | E05.1, E05.2, E05.9 | No | No | No |
| Hypothyroidism^2-4^ | ≥1 recorded diagnosis of hypothyroidism at any time in the past (maximum look-back January 2005; except Norwegian patient data [January 2008]) | E00, E01.8, E02, E03, E89.0 | No | No | No |
| Other disorders of thyroid gland^2-4^ | ≥1 recorded diagnosis of other disorders of thyroid gland at any time in the past (maximum look-back January 2005; except Norwegian patient data [January 2008]) excluding hyperthyroidism and hypothyroidism | E01-E07 (except E05.1, E05.2, E05.9, E01.8, E02, E03) | No | No | No |
| Depression^1-4^ | ≥1 recorded diagnosis of depression OR ≥1 dispensing of antidepressant drugs at any time in the past (maximum look-back January 2005; except for Swedish prescription data [July 2005] and for Norwegian patient data [January 2008]) | F32, F33, F34.1, F43.20 (only Denmark and Finland), F43.22 (only Denmark and Finland) (except F32.8, F33.8) | No | No | N06A |
| Anxiety^1-4^ | ≥1 recorded diagnosis of anxiety OR ≥1 dispensing of benzodiazepines or other anxiolytics at any time in the past (maximum look-back January 2005; except for Swedish prescription data [July 2005] and for Norwegian patient data [January 2008]) | F06.4, F40-F42, F43.0, F43.1 (except F42.4) | No | No | N05BA, N05CD, N05CF02, N05CF01, R06AA02 (not available in Finland and Norway), R06AA09 (not available in Sweden), N05BE01 |
| Sleep disorder^1-4^ | ≥1 recorded diagnosis of sleep disorder at any time in the past (maximum look-back January 2005; except Norwegian patient data [January 2008]) | F51, G47 (except F51.13, G47.4-G47.6) | No | No | No |
| Dementia^1-4^ | ≥1 recorded diagnosis of dementia OR ≥1 dispensing of anti-dementia drugs at any time in the past (maximum look-back January 2005; except for Swedish prescription data [July 2005] and for Norwegian patient data [January 2008]) | F01-F04, F05.1, F06.0, F06.1, F06.8, E75.0, E75.1, E75.2B (only Sweden), E75.2C (only Sweden and Denmark), E75.2W (only Sweden), E75.2D (only Denmark), E75.2E (only Denmark), E75.2 (only Norway and Finland), E75.4, F84.2, G30, G31, G93.7, G93.9 | No | No | N06D |
| Delirium^1-4^ | ≥1 recorded diagnosis of delirium at any time in the past (maximum look-back January 2005; except Norwegian patient data [January 2008]) | F03, F05, F1x.4, G93.4, G92 (except F17.221) | No | No | No |
| Psychosis^1-4^ | ≥1 recorded diagnosis of psychosis at any time in the past (maximum look-back January 2005; except Norwegian patient data [January 2008]) | F03.90, F20, F22-F29, F32.2, F32.3, F33.3, F44.8 (not Finland), F44.88 (Finland only), F84, R44.0, R44.2, R44.3 (except F84.2) | No | No | No |
| **Other Antidiabetic Drug Use** | | | | | |
| N antidiabetic substances at index date^2-4^ | Count of unique antidiabetic substances on the day of initiation of the study drug. Fixed-dose combination products with multiple substances count as multiple drugs, e.g., empagliflozin+metformin results in 2 substances | No | No | A10 | No |
| Naïve new use of antidiabetic drugs^2-4^ | No use of any antidiabetic drug at any time in the past (maximum look-back January 2005; except for Swedish prescription data [July 2005]) preceding index date (including index date) except index (study) drug | No | No | (No history of:) A10 | No |
| Naïve Initiation of the study drug (empagliflozin/any SGLT-2 inhibitor/any DPP-4 inhibitor) as monotherapy^2-4^ | Study drug (empagliflozin/any SGLT-2 inhibitor/ any DPP-4 inhibitor, excluding fixed-dose combinations) initiated as monotherapy, i.e., no filled dispensation of any antidiabetic drugs at any time in the past (maximum look-back January 2005; except for Swedish prescription data [July 2005]) prior to drug initiation (excluding index date)  AND no concomitant initiation of any antidiabetic drugs at index date | No | No | No | Study drug as monotherapy (no fixed-dose combinations): A10BK03, A10BK01, A10BK02, A10BK04, A10BH01, A10BH02, A10BH03, A10BH04, A10BH05 For other antidiabetic drugs: any other A10 code |
| Prior or current use of metformin (regardless of use of use of other antidiabetic agents)^2-4^ | ≥1 filled dispensation of metformin at any time in the past (maximum look-back January 2005; except for Swedish prescription data [July 2005]) preceding index date (including index date) (excluding fixed-dose combinations with metformin and the study drugs; including fixed-dose combinations with metformin and other drugs) | No | No | No | A10BA02, A10BD05, A10BD03 (only in Denmark) |
| Current dual therapy with metformin (without use of other antidiabetic drugs)^2-4^ | ≥2 filled dispensations of metformin in the 183 days prior to index date and current use (i.e., days’ supply overlap) at index date  AND  no filled prescriptions or dispensations of other drugs used in diabetes at any time in the past (maximum look-back January 2005; except for Swedish prescription data [July 2005]) preceding index date (including index date) | No | No | No | A10BA02 For other antidiabetic drugs: A10 |
| Prior or current use of exclusively metformin and no other antidiabetic drugs^2-4^ | ≥1 filled dispensation of metformin at any time in the past (maximum look-back January 2005; except for Swedish prescription data [July 2005]) preceding index date (including index date), without any filled prescription or dispensation of other drugs used in diabetes during the same time period | No | No | No | A10BA02 For other antidiabetic drugs: A10 |
| Concomitant initiation or current use of other antidiabetic drugs^2-4^ | ≥1 filled dispensation of other antidiabetic drugs (including fixed-dose combinations) at index date of the drug of interest  OR  ≥1 filled dispensation of other antidiabetic drugs at any time in the past (maximum look-back January 2005; except for Swedish prescription data [July 2005]) with current use at index date (i.e., days of supply overlap with index date) | No | No | No | A10  AND The drugs/drug classes listed below* separately |
| Past use of other antidiabetic drugs^2-4^ | ≥1 filled prescription/dispensation of other antidiabetic drugs at any time in the past (maximum look-back January 2005; except for Swedish prescription data [July 2005]) prior to index date without current use at index date (i.e., NO overlapping days of supply with index date) | No | No | No | A10 AND The drugs/drug classes listed below* separately |
| Metformin*^1-4^ | ≥1 filled dispensation of metformin at any time in the past (maximum look-back January 2005; except for Swedish prescription data [July 2005]) without current use at index date (i.e., NO overlapping days of supply with index date) | No | No | No | A10BA02, A10BD05, A10BD03 (only in Denmark) |
| Sulfonylureas 2nd generation*^2-4^ | ≥1 filled dispensation of glimepiride, glipizide, glibornuride (also known as glyburide) at any time in the past (maximum look-back January 2005; except for Swedish prescription data [July 2005]) without current use at index date (i.e., NO overlapping days of supply with index date) | No | No | No | A10BB12, A10BB07 (not available in Finland) |
| GLP-1 receptor agonists*^1-4^ | ≥1 filled dispensation of GLP-1 receptor agonists at any time in the past (maximum look-back January 2005; except for Swedish prescription data [July 2005]) without current use at index date (i.e., NO overlapping days of supply with index date) | No | No | No | A10BJ |
| Thiazolidinediones*^1-4^ | ≥1 filled dispensation of thiazolidinediones at any time in the past (maximum look-back January 2005; except for Swedish prescription data [July 2005]) without current use at index date (i.e., NO overlapping days of supply with index date) | No | No | No | A10BG, A10BD03 (only in Denmark), A10BD04 (only in Denmark), A10BD05, A10BD06 (not available in Norway and Sweden) |
| Meglitinides*^2-4^ | ≥1 filled dispensation of meglitinides at any time in the past (maximum look-back January 2005; except for Swedish prescription data [July 2005]) without current use at index date (i.e., NO overlapping days of supply with index date) | No | No | No | A10BX02, A10BX03 (not available in Sweden) |
| Insulin*^2-4^ | ≥1 filled dispensation of insulin at any time in the past (maximum look-back January 2005; except for Swedish prescription data [July 2005]) without current use at index date (i.e., NO overlapping days of supply with index date) | No | No | No | A10A |
| Alpha-glucosidase inhibitors*^2-4^ | ≥1 filled dispensation of alpha-glucosidase inhibitors at any time in the past (maximum look-back January 2005; except for Swedish prescription data [July 2005]) without current use at index date (i.e., NO overlapping days of supply with index date) | No | No | No | A10BF (not available in Finland and Denmark) |
| **Prior Drug Use** | | | | | |
| ACE inhibitor^1-4^ | ≥1 filled dispensation at any time in the past (maximum look-back January 2005; except for Swedish prescription data [July 2005]) | No | No | No | C09A, C09B |
| ARB^1-4^ | ≥1 filled dispensation at any time in the past (maximum look-back January 2005; except for Swedish prescription data [July 2005]) | No | No | No | C09C, C09D |
| Beta blocker^2-4^ | ≥1 filled dispensation at any time in the past (maximum look-back January 2005; except for Swedish prescription data [July 2005]) | No | No | No | C07 |
| Calcium channel blocker^2-4^ | ≥1 filled dispensation at any time in the past (maximum look-back January 2005; except for Swedish prescription data [July 2005]) | No | No | No | C08, C07FB (only in Sweden), C09BB (not available in Sweden), C09DB |
| Thiazides^2-4^ | ≥1 filled dispensation at any time in the past (maximum look-back January 2005; except for Swedish prescription data [July 2005]) | No | No | No | C03A |
| Loop diuretics^1-4^ | ≥1 filled dispensation at any time in the past (maximum look-back January 2005; except for Swedish prescription data [July 2005]) | No | No | No | C03C |
| Other diuretics^1-4^ | ≥1 filled dispensation at any time in the past (maximum look-back January 2005; except for Swedish prescription data [July 2005]) | No | No | No | C03D |
| Nitrates^1-4^ | ≥1 filled dispensation at any time in the past (maximum look-back January 2005; except for Swedish prescription data [July 2005]) | No | No | No | C01DA02, C01DA08 (not available in Sweden), C01DA14, C01EB18 (not available in Sweden and Norway) |
| Other hypertension drugs^2-4^ | ≥1 filled dispensation at any time in the past (maximum look-back January 2005; except for Swedish prescription data [July 2005]) | No | No | No | C02CA04 (not available in Finland), C03DA04, C02CA01 (only in Finland), G04CA03 (not available in Finland), C02AC01 (not available in Norway and Denmark), C02AC02, C02DB02 (not available in Finland and Denmark), C09XA02, C09XA52 (not available in Sweden) |
| Digoxin^1-4^ | ≥1 filled dispensation at any time in the past (maximum look-back January 2005; except for Swedish prescription data [July 2005]) | No | No | No | C01AA05 |
| Valsartan and sacubitril^2-4^ | ≥1 filled dispensation at any time in the past (maximum look-back January 2005; except for Swedish prescription data [July 2005]) | No | No | No | C09DX04 |
| Antiarrhythmic drugs^1-4^ | ≥1 filled dispensation at any time in the past (maximum look-back January 2005; except for Swedish prescription data [July 2005]) | No | No | No | C01B |
| COPD or asthma medications^1-4^ | ≥1 filled dispensation at any time in the past (maximum look-back January 2005; except for Swedish prescription data [July 2005]) | No | No | No | R03AK06, R03AK07, R03AC13, R03AC12, R03AC02, R03CC03 (not available in Finland), R01AX03 (not available in Norway), R03BB01, R03BB04, R03DA04, R03DC03, R03DC01 (only in Finland), R03BB05, R03AL05, R03AC18, R03AC19, R03BB07, R03AL01 (only in Denmark), R03AL02 (not available in Norway), R03AL03, R03AL04, R03AL05, R03AL06, R03AL08, R03AL09 |
| Statin^1-4^ | ≥1 filled dispensation at any time in the past (maximum look-back January 2005; except for Swedish prescription data [July 2005]) | No | No | No | C10AA, C10BA, C10BX |
| PCSK-9 inhibitors^2-4^ | ≥1 filled dispensation at any time in the past (maximum look-back January 2005; except for Swedish prescription data [July 2005]) | No | No | No | C10AX13, C10AX14 |
| Other lipid-lowering drugs, excluding statins^1-4^ | ≥1 filled dispensation at any time in the past (maximum look-back January 2005; except for Swedish prescription data [July 2005]) | No | No | No | C10 |
| Antiplatelet^1-4^ | ≥1 filled dispensation at any time in the past (maximum look-back January 2005; except for Swedish prescription data [July 2005]) | No | No | No | B01AC06, B01AC04, B01AC22, B01AC05 (only in Norway), B01AC07, B01AC23 (only in Sweden), B01AC24 |
| Anticoagulants^1-4^ | ≥1 filled dispensation at any time in the past (maximum look-back January 2005; except for Swedish prescription data [July 2005]) | No | No | No | B01AA03, B01AE07, B01AF01, B01AF02 |
| Heparin and other low-molecular weight heparins^2-4^ | ≥1 filled dispensation at any time in the past (maximum look-back January 2005; except for Swedish prescription data [July 2005]) | No | No | No | B01AB, B01AE07 |
| NSAIDs^2-4^ | ≥1 filled dispensation at any time in the past (maximum look-back January 2005; except for Swedish prescription data [July 2005]) | No | No | No | M01A |
| Oral corticosteroids^1-4^ | ≥1 filled dispensation at any time in the past (maximum look-back January 2005; except for Swedish prescription data [July 2005]) | No | No | No | H02AB10 (only in Norway), H02AB09, H02AB07 (not available in Norway), H02AB06, H02AB04, H02AB08, H02AB02, H02AB01 |
| Bisphosphonates^2-4^ | ≥1 filled dispensation at any time in the past (maximum look-back January 2005; except for Swedish prescription data [July 2005]) | No | No | No | M05BA, M05BB |
| Opioids^2-4^ | ≥1 filled dispensation at any time in the past (maximum look-back January 2005; except for Swedish prescription data [July 2005]) | No | No | No | N02A |
| Antidepressants^2-4^ | ≥1 filled dispensation at any time in the past (maximum look-back January 2005; except for Swedish prescription data [July 2005]) | No | No | No | N06A |
| Antipsychotics^2-4^ | ≥1 filled dispensation at any time in the past (maximum look-back January 2005; except for Swedish prescription data [July 2005]) | No | No | No | N05A |
| Anticonvulsants^1-4^ | ≥1 filled dispensation at any time in the past (maximum look-back January 2005; except for Swedish prescription data [July 2005]) | No | No | No | N03A |
| Lithium^2-4^ | ≥1 filled dispensation at any time in the past (maximum look-back January 2005; except for Swedish prescription data [July 2005]) | No | No | No | N05AN01 |
| Benzodiazepines^2-4^ | ≥1 filled dispensation at any time in the past (maximum look-back January 2005; except for Swedish prescription data [July 2005]) | No | No | No | N05BA, N05CD |
| Other anxiolytics/hypnotics^2-4^ | ≥1 filled dispensation at any time in the past (maximum look-back January 2005; except for Swedish prescription data [July 2005]) | No | No | No | N05CF02, N05CF01, R06AA02 (not available in Finland and Norway), R06AA09 (not available in Sweden), N05BE01 |
| Agents for dementia^2-4^ | ≥1 filled dispensation at any time in the past (maximum look-back January 2005; except for Swedish prescription data [July 2005]) | No | No | No | N06D |
| Antiparkinson agents^2-4^ | ≥1 filled dispensation at any time in the past (maximum look-back January 2005; except for Swedish prescription data [July 2005]) | No | No | No | N04 |
| **Healthcare Resource Utilization** | | | | | |
| Charlson comorbidity score^2-4^ | A patient is deemed to have a Charlson-Quan comorbidity if he or she had been given a diagnosis corresponding to the ICD-code listed in sheet 3.4 at any time in the past (maximum look-back January 2005; except for Swedish prescription data [July 2005] and for Norwegian patient data [January 2008]) | Charlson ME, et a, (1987). "A new method of classifying prognostic comorbidity in longitudinal studies: development and validation". Journal of Chronic Diseases. 40 (5): 373–383. doi:10.1016/0021-9681(87)90171-8. PMID 3558716 | No | No | No |
| Total N distinct diagnosis codes (including index date)^2-4^ | Count of distinct 3-level ICD-10 codes dispensed in the past 12 months | No | No | No | No |
| Number of different medications (including index date)^2-4^ | Count of distinct ATC codes (at 7-digit level) dispensed in the past 12 months | No | No | No | No |
| Any hospitalization^2-4^ | ≥1 hospitalization in the past 12 months | No | No | No | No |
| Any hospitalization within prior 30 days^2-4^ | ≥1 hospitalization in the past 30 day | No | No | No | No |
| Any hospitalization during prior 31-365 days^2-4^ | ≥1 hospitalization during prior 31-365 days | No | No | No | No |
| Number of hospitalizations^2-4^ | Count of hospitalizations in the past 12 months | No | No | No | No |
| Number of hospital days^2-4^ | Sum of hospitalization days in the past 12 months | No | No | No | No |
| Number of emergency department visits^2,4^ | Count of ED visits in the past 12 months (not Norway) | No | No | No | No |
| Number of office visits^2-4^ | Count of visits to physician in specialized outpatient care in the past 12 months | No | No | No | No |
| Endocrinologist visit^2-4^ | ≥1 visit to endocrinologist in the past 12 months (not Norway) | No | No | No | No |
| Endocrinologist visit (30 days prior)^2-4^ | ≥1 visit to endocrinologist in the past 30 days (not Norway) | No | No | No | No |
| Endocrinologist visit (31 to 365 days prior)^2-4^ | ≥1 visit to endocrinologist 31-365 days prior (not Norway) | No | No | No | No |
| Number of endocrinologist visits^2-4^ | Count of visits to endocrinologist in the past 12 months (not Norway) | No | No | No | No |
| Internal medicine/family medicine visit^2-4^ | ≥1 visit to internal medicine/family medicine physician in the past 12 months (not Norway) | No | No | No | No |
| Internal medicine/family medicine visit (30 days prior) ^2-4^ | ≥1 visit to internal medicine/family medicine physician in the past 30 days (not Norway) | No | No | No | No |
| Internal medicine/family medicine visit (31 to 365 days prior) ^2-4^ | ≥1 visit to internal medicine/family medicine physician 31-365 days prior (not Norway) | No | No | No | No |
| Number of internal medicine/family medicine visits^2-4^ | Count of visits to internal medicine/family medicine physician in the past 12 months (not Norway) | No | No | No | No |
| Cardiologist visit^2-4^ | ≥1 visit to cardiologist in the past 12 months (not Norway) | No | No | No | No |
| Cardiologist visit (30 days prior) ^2-4^ | ≥1 visit to cardiologist in the past 30 days (not Norway) | No | No | No | No |
| Cardiologist visit (31 to 365 days prior) ^2-4^ | ≥1 visit to cardiologist 31-365 days prior (not Norway) | No | No | No | No |
| Number of cardiologist visits^2-4^ | Count of visits to cardiologist in the past 12 months (not Norway) | No | No | No | No |
| Electrocardiogram^2-4^ | ≥1 electrocardiogram in the past 12 months | No | Sweden: AF012, AF022-AF025, AF038-AF043, AF070, AV063-AV065.  Norway: FPFE13, FPFE14, FPFE15, FPFE17, FPFE18, FPFE19, FPFE30, FPFE50, FPFE55. Denmark: WHBEK20CY, WHBEK20D2, WHBFAXXXX, WHBGS29CY, WHBGS29KV, WHBGS29XX, WHBGS30KV, WHBGS30XX, WHBHF20A4, WHBHF20CY, WHBHF20D2, WHBHF20XX, WHBHF29XX, WHBHF30XX, ZZ3925, ZZ3926, ZZ3927, ZZ3930, ZZ3931, ZZ4020, ZZ4022, ZZ4023, ZZ4023A, ZZ4023B; Finland: XF400, XF402, XF404, XF405, XF406, XF408, XF409 | No | No |
| Number electrocardiograms received^2-4^ | Count of electrocardiograms in the past 12 months | No | Sweden: AF012, AF022-AF025, AF038-AF043, AF070, AV063-AV065.  Norway: FPFE13, FPFE14, FPFE15, FPFE17, FPFE18, FPFE19, FPFE30, FPFE50, FPFE55. Denmark: WHBEK20CY, WHBEK20D2, WHBFAXXXX, WHBGS29CY, WHBGS29KV, WHBGS29XX, WHBGS30KV, WHBGS30XX, WHBHF20A4, WHBHF20CY, WHBHF20D2, WHBHF20XX, WHBHF29XX, WHBHF30XX, ZZ3925, ZZ3926, ZZ3927, ZZ3930, ZZ3931, ZZ4020, ZZ4022, ZZ4023, ZZ4023A, ZZ4023B. Finland: XF400, XF402, XF404, XF405, XF406, XF408, XF409 | No | No |
| Number of HbA1c tests ordered^2,4^ | Count of HbA1c tests in the past 12 months | No | No | Codes are available below | No |
| Number of BUN tests ordered^2,4^ | Count of BUN tests in the past 12 months | No | No | Codes are available below | No |
| Number of glucose tests ordered^2^ | Count of glucose tests in the past 12 months | No | No | Codes are available below | No |
| Number of lipid tests ordered^2,4^ | Count of lipid tests in the past 12 months | No | No | Codes are available below | No |
| Number of creatinine tests ordered^2,4^ | Count of creatinine tests in the past 12 months | No | No | Codes are available below | No |
| Number of tests for microalbuminuria^2,4^ | Count of microalbuminuria tests in the past 12 months | No | No | Codes are available below | No |
| ^1^Included in propensity score matching for country-level analyses in Denmark  ^2^Included in propensity score matching for country-level analyses in Finland  ^3^Included in propensity score matching for country-level analyses in Norway  ^4^Included in propensity score matching for country-level analyses in Sweden  * Past use.  Abbreviations: ACE=Angiotensin-converting-enzyme; AF=Atrial fibrillation; ARB=Angiotensin II receptor blockers; ATC=Anatomic Therapeutic Chemical classification; BNP=B-type Natriuretic Peptide; BUN=Blood urea nitrogen; BNP=B-type natriuretic peptide; CABG=coronary artery bypass grafting; CHF=Congestive heart failure; COPD=Chronic obstructive pulmonary disease; CV=Cardiovascular; DPP-4=Dipeptidyl peptidase-4; DRG=Diagnosis related groups; ED=Emergency department; GFR=Glomerular filtration rate; GGT=Gamma-glutamyl transferase; GLP-1=Glucagon-like peptide-1; HbA1c=Glycated hemoglobin; HCRU=Healthcare resource utilization; HDL=High-density lipoprotein; ICD-10=International Statistical Classification of Diseases and Related Health Problems - Tenth Revision; LDL=Low-density lipoprotein; MI=Myocardial infarction; NA=Not applicable; NCSP=NOMESCO (Nordic Medico-Statistical Committee) Classification of Surgical Procedures; NSAID=Non-steroidal anti-Inflammatory drugs; PCSK-9=Proprotein convertase subtilisin/kexin type 9; PTCA = percutaneous transluminal coronary angioplasty; SGLT-2=Sodium-glucose cotransporter 2; SGOT=Serum glutamic oxaloacetic transaminase; SGPT=Serum glutamic pyruvic transaminase; T2D=Type 2 diabetes; UACR=Urine albumin-to-creatinine ratio. | | | | | |
