# Supplementary material for: Empagliflozin Use Is Associated With Lower Risk of All-Cause Mortality, Hospitalization for Heart Failure, and End-Stage Renal Disease Compared to DPP-4i in Nordic Type 2 Diabetes Patients: Results From the EMPRISE (Empagliflozin Comparative Effectiveness and Safety) Study
Source: J Diabetes Res. 2024 Oct 12;2024:6142211. doi: 10.1155/2024/6142211 (PMC11490347; doi:10.1155/2024/6142211)
Supplement: Supporting Information — Additional supporting information can be found online in the Supporting Information section. The supporting information provides additional details regarding (A) the characteristics of data sources from the four Nordic countries; (B) the definitions of exposure periods; (C) definitions of covariates, propensity score variables, and laboratory values; (D) definitions for the study outcomes; and (E) the baseline patient characteristics by country and study subgroup. Description of the data sources in four Nordic countries. This study is based on several nationwide data sources of observational data (national registers) in four Nordic countries, namely, Denmark, Finland, Norway, and Sweden. Three types of national registers were used in this study for all Nordic countries: patient registers, prescription registers, and cause of death registers. Additionally, national, or regional registers containing laboratory values and lifestyle factors were utilized. Patients with dispensations of empagliflozin, or any dipeptidyl peptidase-4 inhibitor (DPP-4i), were identified in the prescription registers. The identified population was then linked to the other registers used in this study. All data was deidentified, and unique individual patient identification numbers were available for all data sources which allowed for extensive linkage between data sets in each country. For Finland, data on socioeconomic status was also extracted. Due to Norwegian regulations and the pseudonymization of the prescription register, identification of patients was a two-step process: first by diagnosis (at any position) in inpatient, outpatient, or primary care and then by adding prescription data to identified subjects. In this country, International Classification of Primary Care, 2nd edition (ICPC-2) codes were used to identify type 2 diabetes (T2D) patients in primary care (the “Kontroll og utbetaling av helserefusjoner” (KUHR) register) and the International Classification of Diseases and Rela [file 6142211.f1.zip › Supplementary Table 3.docx]

Table 3. Definitions of laboratory values

| **Laboratory variable** | **Definition** | **Relevant unit** | **Data source** | **Laboratory variable(s) available in the data source** | **Variable name/code (Danish NPU or Finnish national codes)** | **Unit in the available source** | **Suggested choice of laboratory variable if several are available** | **Transformation from unit available to relevant unit** |
| --- | --- | --- | --- | --- | --- | --- | --- | --- |
| HbA1c^1,2,4^ | Most recent HbA1c value (past 12 months) | mmol/mol | NDR | HbA1c | hba1c | mmol/mol (IFCC) | NA | As given |
| LDL level^1,2,4^ | Most recent LDL value, plasma or serum (past 12 months) | mg/dl | NDR | LDL | ldl | mmol/l | NA | mmol/L x 38.67 = mg/dL |
| HDL level^1,2,4^ | Most recent HDL value, plasma or serum (past 12 months) | mg/dl | NDR | HDL | hdl | mmol/l | NA | mmol/L x 38.67= mg/dL |
| Total cholesterol^1,2,4^ | Most recent total cholesterol value (past 12 months) | mg/dl | NDR | Cholesterol | kolesterol | mmol/l | NA | mmol/L x 38.67 = mg/dL |
| Triglyceride level^1,2,4^ | Most recent triglyceride level value (past 12 months) | mg/dl | NDR | Triglycerides | triglycerider | mmol/l | NA | mmol/L x 88.57 = mg/dL |
| Creatinine^1,2,4^ | Most recent creatinine value (past 12 months) | mg/dl | NDR | Creatinine adjusted albumin | kreatinin | mg/mmol | NA | mg/mmol x 0.0113 = mg/dL |
| Estimated Glomerular Filtration Rate (eGFR)^1,2,4^ | Most recent GFR value (past 12 months) | mL/min/1.73m2 | NDR | Creatinine (plasma/serum) will be used to calculate eGFR based on the CKD-EPI equation – MDRD equation – or both | kreatinin | NA | NA | eGFR=141*min(Cr/κ, 1)^α^ *max(Cr/κ, 1)-1.209*0.993Age [*1.018 if female] where Cr=creatinine, κ is 0.7 for female and 0.9 for male, α is -0.329 for female and -0.411 for male |
| BUN (Blood Urea Nitrogen)^2^ | Most recent BUN value (past 12 months) | mmol/L | Not available | NA | NA | NA | NA | NA |
| BNP (B-type Natriuretic Peptide)^2^ | Most recent BNP value (past 12 months) | ng/L | Not available | NA | NA | NA | NA | NA |
| NT-proBNP^2^ | Most recent NT-proBNP value (past 12 months) | ng/L | Not available | NA | NA | NA | NA | NA |
| SGOT (AST)^2^ | Most recent SGOT value (past 12 months) | U/L | Not available | NA | NA | NA | NA | NA |
| SGPT (ALT)^1,2^ | Most recent SGPT value (past 12 months) | U/L | Not available | NA | NA | NA | NA | NA |
| GGT^2^ | Most recent GGT value (past 12 months) | U/L | Not available | NA | NA | NA | NA | NA |
| Alkaline phosphatase^1,2^ | Most recent alkaline phosphatase value (past 12 months) | U/L | Not available | NA | NA | NA | NA | NA |
| UACR (Urine albumin-to-creatinine ratio)^1,2^ | Most recent UACR value (past 12 months) | mg/mmol | NDR | Urine albumin/creatinine ratio | uAlbCreatinine | mg/mmol | NA | NA |
| Urine Protein^2^ | Most recent urine protein value (past 12 months) | g/l | Not available | NA | NA | NA | NA | NA |
| Potassium^12^ | Most recent potassium value (past 12 months) | mmol/l | Not available | NA | NA | NA | NA | NA |
| Calcium^2^ | Most recent calcium value (past 12 months) | mmol/l | Not available | NA | NA | NA | NA | NA |
| Albuminuria^2^ | Most recent albuminuria measure categorized as "mikro albuminuria", "makro albuminuria" or "no albuminuria" in the past 12 months | Categorical variable:  Micro albuminuria Macro albuminuria Normal albuminuria | NDR | Albuminuria given in 3 categories "mikro albuminuria", "macro albuminuria" or "normal value" | albuminuria | Category | NA | NA |
| Total glucose^1,2^ | Most recent total glucose value (past 12 months) | mmol/l | NDR | Plasma glucose (highest value) | incFDPG | mmol/l | NA | NA |
| Phosphorus^2^ | Most recent phosphorus value (past 12 months) | mmol/l | Not available | NA | NA | NA | NA | NA |
| ^1^ Included in propensity score matching for country-level analyses in Denmark  ^2^ Included in propensity score matching for country-level analyses in Finland  ^3^ Included in propensity score matching for country-level analyses in Norway  ^4^ Included in propensity score matching for country-level analyses in Sweden  BUN = Blood urea nitrogen; BNP = B-type natriuretic peptide; CKD-EPI equation = Chronic Kidney Disease Epidemiology Collaboration equation; HbA1c = Glycated hemoglobin; HDL = High-density lipoprotein level; LDL = Low-density lipoprotein level; MDRD equation = Modification of Diet in Renal Disease equation | | | | | | | | |
