# Supplementary material for: Empagliflozin Use Is Associated With Lower Risk of All-Cause Mortality, Hospitalization for Heart Failure, and End-Stage Renal Disease Compared to DPP-4i in Nordic Type 2 Diabetes Patients: Results From the EMPRISE (Empagliflozin Comparative Effectiveness and Safety) Study
Source: J Diabetes Res. 2024 Oct 12;2024:6142211. doi: 10.1155/2024/6142211 (PMC11490347; doi:10.1155/2024/6142211)
Supplement: Supporting Information — Additional supporting information can be found online in the Supporting Information section. The supporting information provides additional details regarding (A) the characteristics of data sources from the four Nordic countries; (B) the definitions of exposure periods; (C) definitions of covariates, propensity score variables, and laboratory values; (D) definitions for the study outcomes; and (E) the baseline patient characteristics by country and study subgroup. Description of the data sources in four Nordic countries. This study is based on several nationwide data sources of observational data (national registers) in four Nordic countries, namely, Denmark, Finland, Norway, and Sweden. Three types of national registers were used in this study for all Nordic countries: patient registers, prescription registers, and cause of death registers. Additionally, national, or regional registers containing laboratory values and lifestyle factors were utilized. Patients with dispensations of empagliflozin, or any dipeptidyl peptidase-4 inhibitor (DPP-4i), were identified in the prescription registers. The identified population was then linked to the other registers used in this study. All data was deidentified, and unique individual patient identification numbers were available for all data sources which allowed for extensive linkage between data sets in each country. For Finland, data on socioeconomic status was also extracted. Due to Norwegian regulations and the pseudonymization of the prescription register, identification of patients was a two-step process: first by diagnosis (at any position) in inpatient, outpatient, or primary care and then by adding prescription data to identified subjects. In this country, International Classification of Primary Care, 2nd edition (ICPC-2) codes were used to identify type 2 diabetes (T2D) patients in primary care (the “Kontroll og utbetaling av helserefusjoner” (KUHR) register) and the International Classification of Diseases and Rela [file 6142211.f1.zip › Supplementary Table 4.docx]

Table 4. Definitions of study outcomes

| **Outcome** | **Definition** | **Information used in identifying the outcome** | | | |
| --- | --- | --- | --- | --- | --- |
|  |  | **ICD-10 codes** | **NCSP/non-surgical procedure codes (any position)** | **Laboratory measurements** | **ATC codes** |
| **HHF** | Primary diagnosis associated with hospital admission (>1 day) | I50, I09.0 (only Sweden), I09.9 (only Sweden and Finland), I09.8 (only Norway and Finland), I09.9A (only Denmark), I11.0, I13.0, I13.2 | No | No | No |
| **ACM** | Death by any cause | No | No | No | No |
| **MI** | Myocardial infarction | I21, I22 | No | No | No |
| **Stroke** | Stroke | I60, I61, I63, I64, I67.8 | No | No | No |
| **CVM** | Death from any cardiovascular condition, OR Death from diabetes with vascular complication, OR  Death within 30 days of a CV event hospitalization (including heart failure, MI, coronary revascularization procedure) | Any CV: I00-I99, HF: I50, I09.0 (only Sweden), I09.9 (only Sweden and Finland), I09.8 (only Norway and Finland), I09.9A (only Denmark), I11.0, I13.0, I13.2, MI: I21-I22, Stroke: I60, I61, I63, I64 | Coronary revascularization procedure:  FNA00, FNA10, FNA20, FNA96, FNB00, FNB20, FNB96, FNC10, FNC20, FNC30, FNC40, FNC50, FNC60, FNC96, FND10, FND20, FND96, FNE00, FNE10, FNE20, FNE96, FNF00, FNF10, FNF20, FNF30, FNF96, FNG00, FNG02, FNG05, FNG06, FNG10, FNG30, FNG96 | No | No |
| **ESRD** | eGFR<15ml/min at least 2 measurements separated by at least 30 days (but no more than 12 months),  OR  ≥2 of the following diagnosis codes, separated by at least 30 days:  Diagnoses: N18.5, N18.6, Z99.2  OR  Kidney transplant, defined as ≥1 of the diagnosis or procedure codes (inpatient or outpatient),  OR  Use of ESRD related drugs, defined as ≥1 filled prescriptions with the ATC codes.  The cut-off date for look-back period was January 2005 due to data availability, except for Swedish prescription data (July 2005) and Norwegian patient data (January 2008). | N18.5 (not available in Finland), N18.6 (only available in Finland), Z99.2, Z94.0, T86.1 | KAS00, KAS10, KAS40 | Yes | B03XA01, V03AE07, V03AE04, V03AE03, V03AE02, V03AE05, V03AE08 |
| ACM=all-cause mortality; ATC=Anatomical Therapeutic Chemical code; CV=cardiovascular; CVM=cardiovascular mortality; ESRD=end-stage-renal-disease; eGFR=estimated glomerular filtration rate; MI=myocardial infarction; NCSP=NOMESCO (Nordic Medico-Statistical Committee) Classification of Surgical Procedures; ICD-10=the International Classification of Diseases and Related Health Problems, 10th revision. | | | | | |
