# Supplementary material for: Empagliflozin Use Is Associated With Lower Risk of All-Cause Mortality, Hospitalization for Heart Failure, and End-Stage Renal Disease Compared to DPP-4i in Nordic Type 2 Diabetes Patients: Results From the EMPRISE (Empagliflozin Comparative Effectiveness and Safety) Study
Source: J Diabetes Res. 2024 Oct 12;2024:6142211. doi: 10.1155/2024/6142211 (PMC11490347; doi:10.1155/2024/6142211)
Supplement: Supporting Information — Additional supporting information can be found online in the Supporting Information section. The supporting information provides additional details regarding (A) the characteristics of data sources from the four Nordic countries; (B) the definitions of exposure periods; (C) definitions of covariates, propensity score variables, and laboratory values; (D) definitions for the study outcomes; and (E) the baseline patient characteristics by country and study subgroup. Description of the data sources in four Nordic countries. This study is based on several nationwide data sources of observational data (national registers) in four Nordic countries, namely, Denmark, Finland, Norway, and Sweden. Three types of national registers were used in this study for all Nordic countries: patient registers, prescription registers, and cause of death registers. Additionally, national, or regional registers containing laboratory values and lifestyle factors were utilized. Patients with dispensations of empagliflozin, or any dipeptidyl peptidase-4 inhibitor (DPP-4i), were identified in the prescription registers. The identified population was then linked to the other registers used in this study. All data was deidentified, and unique individual patient identification numbers were available for all data sources which allowed for extensive linkage between data sets in each country. For Finland, data on socioeconomic status was also extracted. Due to Norwegian regulations and the pseudonymization of the prescription register, identification of patients was a two-step process: first by diagnosis (at any position) in inpatient, outpatient, or primary care and then by adding prescription data to identified subjects. In this country, International Classification of Primary Care, 2nd edition (ICPC-2) codes were used to identify type 2 diabetes (T2D) patients in primary care (the “Kontroll og utbetaling av helserefusjoner” (KUHR) register) and the International Classification of Diseases and Rela [file 6142211.f1.zip › Supplementary Table 5.docx]

Table 5. Baseline Characteristics for empagliflozin and DPP-4i Subcohorts After PS-Matching

| **Attributes** | **Denmark** | | | **Finland** | | | **Norway** | | | **Sweden** | | | **Total** | |
| --- | --- | --- | --- | --- | --- | --- | --- | --- | --- | --- | --- | --- | --- | --- |
|  | **EMPA** | **DDP-4i** | **Std**  **Diff** | **EMPA** | **DDP-4i** | **Std**  **Diff** | **EMPA** | **DDP-4i** | **Std**  **Diff** | **EMPA** | **DDP-4i** | **Std**  **Diff** | **EMPA** | **DDP-4i** |
| **No∙ of patients (N)** | 9765 | 9765 | - | 11801 | 11801 | - | 6344 | 6344 | - | 15785 | 15785 | - | 43695 | 43695 |
| **Age at index (years)** |  |  |  |  |  |  |  |  |  |  |  |  |  |  |
| Mean | 61.4 | 61.3 | 0.01 | 62.5 | 62.5 | 0.00 | 60.8 | 60.8 | 0.01 | 63.5 | 63.4 | 0.01 | 60.8-63.5 | 60.8-63.4 |
| SD | 11.6 | 12.8 | - | 11.1 | 12.0 |  | 11.9 | 12.4 | - | 10.9 | 11.5 | - | - | - |
| Median | 62.1 | 61.6 | - | 63 | 64 |  | 61 | 62 | - | 65 | 65 | - | - | - |
| Q1; Q3 | 53.6; 70.0 | 52.4; 70.7 | - | 55.0; 70.0 | 55.0; 71.0 |  | 53.0; 69.0 | 53.0; 70.0 | - | 56.0; 71.0 | 56.0; 71.0 | - | - | - |
| Min; Max | - | - | - | 18.0; 99.0 | 18.0; 103.0 |  | 19.0; 99.0 | 18.0; 96.0 | - | 18.0; 95.0 | 18.0; 96.0 | - | - | - |
| 18-54 | - | - | - | 2797 (23.7) | 2803 (23.8) |  | 1905 (30.0) | 1859 (29.3) | - | 3289 (20.8) | 3350 (21.2) | - | - | - |
| 55-64 | - | - | - | 3526 (29.9) | 3452 (29.3) |  | 1910 (30.1) | 1935 (30.5) | - | 4490 (28.4) | 4489 (28.4) | - | - | - |
| 65-74 | - | - | - | 3910 (33.1) | 3994 (33.8) |  | 1779 (28.0) | 1802 (28.4) | - | 5700 (36.1) | 5679 (36.0) | - | - | - |
| 75+ | - | - | - | 1568 (13.3) | 1552 (13.2) |  | 750 (11.8) | 748 (11.8) | - | 2306 (14.6) | 2267 (14.4) | - | - | - |
| **Sex** | | | | | | | | | | | | | | |
| Female, n (%) | 3647 (37.3) | 3720 (38.1) | 0.02 | 4880 (41.4) | 4920 (41.7) | 0.01 | 2287 (36.0) | 2324 (36.6) | 0.01 | 5583 (35.4) | 5530 (35.0) | 0.01 | 16397 (37.5) | 16494 (37.8) |
| Male, n (%) | 6118 (62.7) | 6045 (61.9) | - | 6921 (58.6) | 6881 (58.3) |  | 4057 (64.0) | 4020 (63.4) | - | 10202 (64.6) | 10255 (65.0) | - | 27298 (62.5) | 27201 (62.3) |
| **Laboratory values** | | | | | | | | | | | | | | |
| **HbA_1c_ (mmol/mol)** | | | | | | | | | | | | | | |
| Mean | 52.5 | 52.4 | 0.00 | 57.8 | 55.8 | 0.11 | - | - | - | 65.3 | 65.3 | 0.01 | - | - |
| SD | 33.4 | 33.6 | - | 18.1 | 17.5 | - | - | - | - | 15.3 | 15.8 | - | - | - |
| Median | 60 | 59 | - | 53 | 51 | - | - | - | - | 62 | 62 | - | - | - |
| Q1; Q3 | 41.0; 73.0 | 41.0; 72.0 | - | 45.0; 65.0 | 44.0; 62.0 | - | - | - | - | 55.0; 73.0 | 54.0; 73.0 | - | - | - |
| Min; Max | - | - | - | 25.0; 168.0 | 20.0; 165.0 | - | - | - | - | 28.0; 153.0 | 25.0; 166.0 | - | - | - |
| Missing | - | - | - | 6750 (57.2) | 6677 (56.6) | - | - | - | - | - | - | - | - | - |
| **Total cholesterol (mg/dL)** | | | | | | | | | | | | | | |
| Mean | 123.7 | 123.7 | 0.01 | 178.5 | 178.2 | 0.01 | - | - | - | 174.2 | 173.8 | 0.00 | - | - |
| SD | 81.2 | 81.2 | - | 46.3 | 45.5 | - | - | - | - | 45.1 | 44.6 | - | - | - |
| Median | 143.1 | 143.1 | - | 174.0 | 174.0 | - | - | - | - | 166.3 | 166.3 | - | - | - |
| Q1; Q3 | 0.0; 177.9 | 0.0; 177.9 | - | 147.0; 205.0 | 147.0; 205.0 | - | - | - | - | 143.1; 201.1 | 143.1; 201.1 | - | - |  |
| Min; Max | - | - | - | 61.9; 773.4 | 58.0; 576.2 | - | - | - | - | 46.4; 549.1 | 50.3; 657.4 | - | - | - |
| Missing | - | - | - | 6570 (55.7) | 6573 (55.7) | - | - | - | - | - | - | - | - | - |
| **Low-density lipoprotein level (mg/dL)** | | | | | | | | | | | | | | |
| Mean | 58.0 | 58.0 | 0.01 | 106.2 | 106.1 | 0.00 | - | - | - | 95.8 | 95.6 | 0.00 | - | - |
| SD | 46.4 | 50.3 | - | 38.6 | 38.5 | - | - | - | - | 37.7 | 37.6 | - | - | - |
| Median | 61.9 | 61.9 | - | 100.5 | 100.5 | - | - | - | - | 89.3 | 89.3 | - | - | - |
| Q1; Q3 | 0.0; 88.9 | 0.0; 88.9 | - | 77.3; 131.5 | 77.3; 129.9 | - | - | - | - | 69.2; 117.6 | 68.5; 117.9 | - | - | - |
| Min; Max | - | - | - | 7.7; 309.4 | 7.7; 282.3 | - | - | - | - | 8.9; 321.0 | 8.1; 357.7 | - | - | - |
| Missing | - | - | - | 6714 (56.9) | 6673 (56.5) | - | - | - | - | - | - | - | - | - |
| **High-density lipoprotein level (mg/dL)** | | | | | | | | | | | | | | |
| Mean | 30.9 | 34.8 | 0.01 | 47.1 | 47.5 | 0.03 | - | - | - | 44.9 | 44.8 | 0.01 | - | - |
| SD | 23.2 | 23.2 | - | 13.5 | 13.9 | - | - | - | - | 13.0 | 12.9 | - | - | - |
| Median | 38.7 | 38.7 | - | 45.2 | 45.6 | - | - | - | - | 42.5 | 42.5 | - | - | - |
| Q1; Q3 | 0.0; 46.4 | 0.0; 46.4 | - | 37.9; 54.1 | 37.9; 54.9 | - | - | - | - | 34.8; 50.3 | 34.8; 50.3 | - | - | - |
| Min; Max | - | - | - | 3.9; 141.2 | 8.5; 205.0 | - | - | - | - | 7.7; 193.4 | 7.7; 170.2 | - | - | - |
| Missing | - | - | - | 6589 (55.8) | 6605 (56.0) | - |  | - | - |  |  |  | - | - |
| **Triglyceride level (mg/dL)** | | | | | | | | | | | | | | |
| Mean | 168.3 | 168.3 | 0.00 | 188.3 | 183.0 | 0.04 | - | - | - | 192.6 | 191.5 | 0.00 | - | - |
| SD | 203.7 | 194.9 | - | 152.1 | 141.8 | - | - | - | - | 152.9 | 137.1 | - | - | - |
| Median | 141.7 | 141.7 | - | 155.9 | 154.1 | - | - | - | - | 159.4 | 159.4 | - | - | - |
| Q1; Q3 | 0.0; 230.3 | 0.0; 230.3 | - | 115.1; 218.8 | 109.8; 212.6 | - | - | - | - | 115.1; 221.4 | 115.1; 221.4 | - | - | - |
| Min; Max | - | - | - | 29.2; 4724.3 | 31.0; 3099.1 | - | - | - | - | 8.9; 3542.8 | 26.6; 2391.4 | - | - | - |
| Missing | - | - | - | 6580 (55.8) | 6595 (55.9) | - | - | - | - | - | - | - | - | - |
| **Estimated Glomerular Filtration Rate (mL/min/1.73 m^2^)** | | | | | | | | | | | | | | |
| Mean | 80.4 | 80.7 | 0.02 | 87.9 | 85.2 | 0.14 | - | - | - | 87.3 | 87.2 | 0.02 | - | - |
| SD | 16.3 | 18.9 | - | 17.1 | 21.8 | - | - | - | - | 17.5 | 19.3 | - | - | - |
| Median | 88.0 | 90.0 | - | 89.7 | 89.3 | - | - | - | - | 89.4 | 90.4 | - | - | - |
| Q1; Q3 | 72.0; 90.0 | 73.0; 90.0 | - | 76.4; 99.5 | 71.9; 100.6 | - | - | - | - | 76.1; 98.9 | 75.4; 100.4 | - | - | - |
| Min; Max | - | - | - | 19.3; 157.2 | 12.4; 175.5 | - | - | - | - | 14.8; 216.2 | 13.9; 201.5 | - | - | - |
| Missing | - | - | - | 5940 (50.3) | 5825 (49.4) | - | - | - | - | - | - | - | - | - |
| **Creatinine (mg/dL)** |  |  |  |  |  |  |  |  |  |  |  |  | - | - |
| Mean | 0.7 | 0.7 | 0.01 | 0.8 | 0.9 | 0.21 | - | - | - | 0.8 | 0.8 | 0.01 | - | - |
| SD | 0.4 | 0.4 | - | 0.2 | 0.3 | - | - | - | - | 0.2 | 0.2 | - | - | - |
| Median | 0.7 | 0.7 | - | 0.8 | 0.8 | - | - | - | - | 0.8 | 0.8 | - | - | - |
| Q1; Q3 | 0.5; 0.9 | 0.5; 0.9 | - | 0.7; 0.9 | 0.7; 1.0 | - | - | - | - | 0.7; 1.0 | 0.7; 1.0 | - | - | - |
| Min; Max | - | - | - | 0.4; 3.3 | 0.3; 3.6 | - | - | - | - | 0.1; 4.4 | 0.1; 3.2 | - | - | - |
| Missing | - | - | - | 5934 (50.3) | 5823 (49.3) | - | - | - | - | - | - | - | - | - |
| **Comorbidities and Other Medical Conditions n (%)** | | | | | | | | | | | | | | |
| Diabetic retinopathy | 493  (5.0) | 449  (4.6) | 0.02 | 412  (3.5) | 396 (3.4) | 0.01 | 557  (8.8) | 568  (9.0) | 0.01 | 1679 (10.6) | 1602 (10.1) | 0.02 | 3141 (7.2) | 3015 (6.9) |
| Diabetes with other ophthalmic manifestations | - | - | - | 148  (1.3) | 138  (1.2) | 0.01 | 473  (7.5) | 472  (7.4) | 0.00 | 1444 (9.1) | 1384 (8.8) | 0.01 | - | - |
| Retinal detachment, vitreous hemorrhage, vitrectomy | 59  (0.6) | 68  (0.7) | 0.01 | 159  (1.3) | 166 (1.4) | 0.01 | 71  (1.1) | 66  (1.0) | 0.01 | 269  (1.7) | 278  (1.8) | 0.00 | 558 (1.3) | 578 (1.3) |
| Retinal laser coagulation therapy | 35  (0.4) | 41  (0.4) | 0.01 | 33  (0.3) | 31  (0.3) | 0.00 | 14  (0.2) | 11  (0.2) | 0.01 | 88  (0.6) | 98  (0.6) | 0.01 | 170 (0.4) | 181 (0.4) |
| Diabetic neuropathy | 373  (3.8) | 319  (3.3) | 0.03 | 115  (1.0) | 124  (1.1) | 0.01 | 91  (1.4) | 95  (1.5) | 0.01 | 212  (1.3) | 212  (1.3) | 0.00 | 791 (1.8) | 750 (1.7) |
| Diabetic nephropathy | 307  (3.1) | 271  (2.8) | 0.02 | 101  (0.9) | 113  (1.0) | 0.01 | 61  (1.0) | 57  (0.9) | 0.01 | 159  (1.0) | 160  (1.0) | 0.00 | 628 (1.4) | 601 (1.4) |
| Hypoglycemia | - | - | - | 34  (0.3) | 36  (0.3) | 0.00 | 13  (0.2) | 13  (0.2) | 0.00 | 132  (0.8) | 122  (0.8) | 0.01 | - | - |
| Hyperglycemia | 167  (1.7) | 158  (1.6) | 0.01 | 89  (0.8) | 94  (0.8) | 0.00 | 20  (0.3) | 23  (0.4) | 0.01 | 502  (3.2) | 496  (3.1) | 0.00 | 778 (1.8) | 771 (1.8) |
| Disorders of fluid electrolyte and acid-base balance | 248  (2.5) | 246  (2.5) | 0.00 | 184  (1.6) | 178  (1.5) | 0.00 | 43  (0.7) | 36  (0.6) | 0.01 | 285  (1.8) | 262  (1.7) | 0.01 | 760 (1.7) | 722 (1.7) |
| Diabetic ketoacidosis / Lactic acidosis | - | - | - | 332  (2.8) | 339  (2.9) | 0.00 | 278  (4.4) | 289  (4.6) | 0.01 | 101  (0.6) | 88  (0.6) | 0.01 | - | - |
| Hyperglycemic hyperosmolar non-ketotic syndrome (HHNS) | - | - | - | 314  (2.7) | 318  (2.7) | 0.00 | 265  (4.2) | 275  (4.3) | 0.01 | 42  (0.3) | 38  (0.2) | 0.01 | - | - |
| Diabetes and peripheral vascular disease | 182  (1.9) | 181  (1.9) | 0.00 | 138  (1.2) | 141  (1.2) | 0.00 | 52  (0.8) | 50  (0.8) | 0.00 | 137  (0.9) | 151  (1.0) | 0.01 | 509 (1.2) | 523 (1.2) |
| Diabetic foot | 126  (1.3) | 132  (1.4) | 0.01 | 0 (0.0) | 0 (0.0) | - | - | - | - | 113  (0.7) | 113  (0.7) | 0.00 | 239 (0.6) | 245 (0.7) |
| Gangrene | 22  (0.2) | 21  (0.2) | 0.00 | 6  (0.1) | 7  (0.1) | 0.00 | <5 | <5 | 0.00 | <5 | 0 (0.0) | 0.01 | 30-36 (0.1-0.1) | 29-32 (0.1-0.1) |
| Lower-extremity amputation | <5 | <5 | 0.00 | <5 | <5 | 0.00 | <5 | <5 | 0.02 | 7 (0.0) | 5 (0.0) | 0.01 | 10-19 (0.0-0.0) | 8-17 (0.0-0.0) |
| Osteomyelitis | 13  (0.1) | 19  (0.2) | 0.02 | 42  (0.4) | 35  (0.3) | 0.01 | 19  (0.3) | 21  (0.3) | 0.01 | 63  (0.4) | 50  (0.3) | 0.01 | 137 (0.3) | 125 (0.3) |
| Skin infections | 358  (3.7) | 363  (3.7) | 0.00 | 441  (3.7) | 420  (3.6) | 0.01 | 227  (3.6) | 221  (3.5) | 0.01 | 756  (4.8) | 769  (4.9) | 0.00 | 1782 (4.1) | 1773 (4.1) |
| Erectile dysfunction | 64  (0.7) | 61  (0.6) | 0.00 | 29  (0.2) | 30  (0.3) | 0.00 | 27  (0.4) | 24  (0.4) | 0.01 | 167  (1.1) | 176  (1.1) | 0.01 | 287 (0.7) | 291 (0.7) |
| Diabetes with unspecified complication | 848  (8.7) | 791  (8.1) | 0.02 | 739  (6.3) | 755  (6.4) | 0.01 | 232  (3.7) | 252  (4.0) | 0.02 | 808  (5.1) | 824  (5.2) | 0.00 | 2627 (6.0) | 2622 (6.0) |
| Diabetes mellitus without mention of complications | 4476 (45.8) | 4415 (45.2) | 0.01 | 3354 (28.4) | 3413 (28.9) | 0.01 | 3489 (55.0) | 3533 (55.7) | 0.01 | 7650 (48.5) | 7793 (49.4) | 0.02 | 18969 (43.4) | 19154 (43.8) |
| T2D diagnosis in national register | 464  (4.8) | 431  (4.4) | 0.02 | - | - | - | - | - | - | - | - | - | - | - |
| Diabetic keto/lactateacidosis | 35  (0.4) | 31  (0.3) | 0.01 | - | - | - | - | - | - | - | - | - | - | - |
| Mild hypoglycemia | 10  (0.1) | 8  (0.1) | 0.01 | - | - | - | - | - | - | - | - | - | - | - |
| Severe hypoglycemia | 86  (0.9) | 86  (0.9) | 0.00 | - | - | - | - | - | - | - | - | - | - | - |
| Hypertension | 2607 (26.7) | 2514 (25.7) | 0.02 | 3040 (25.8) | 3071 (26.0) | 0.01 | 390 (6.1) | 391 (6.2) | 0.00 | 4343 (27.5) | 4289 (27.2) | 0.01 | 10380 (23.8) | 10265 (23.5) |
| Hyperlipidemia | 1306 (13.4) | 1233 (12.6) | 0.02 | 1925 (16.3) | 1916 (16.2) | 0.00 | 783 (12.3) | 794 (12.5) | 0.01 | 3109 (19.7) | 3134 (19.9) | 0.00 | 7123 (16.3) | 7077 (16.2) |
| Ischemic heart disease | 951 (9.7) | 885 (9.1) | 0.02 | 1772 (15.0) | 1718 (14.6) | 0.01 | 1337 (21.1) | 1310 (20.6) | 0.01 | 3478 (22.0) | 3446 (21.8) | 0.00 | 7538 (17.3) | 7359 (16.8) |
| Acute MI | 414 (4.2) | 373 (3.8) | 0.02 | 595 (5.0) | 563 (4.8) | 0.01 | 580 (9.1) | 531 (8.4) | 0.03 | 1758 (11.1) | 1713 (10.9) | 0.01 | 3347 (7.7) | 3180 (7.3) |
| Acute coronary syndrome/unstable angina | - | - | - | 1275 (10.8) | 1224 (10.4) | 0.01 | 1054 (16.6) | 1017 (16.0) | 0.02 | 3060 (19.4) | 3031 (19.2) | 0.00 | - | - |
| Old MI | 204 (2.1) | 167 (1.7) | 0.03 | 419 (3.6) | 394 (3.3) | 0.01 | 288 (4.5) | 276 (4.4) | 0.01 | 1681 (10.6) | 1659 (10.5) | 0.00 | 2592 (5.9) | 2496 (5.7) |
| Stable angina | 634 (6.5) | 607 (6.2) | 0.01 | 702 (5.9) | 679 (5.8) | 0.01 | 554 (8.7) | 532 (8.4) | 0.01 | 1659 (10.5) | 1677 (10.6) | 0.00 | 3549 (8.1) | 3495 (8.0) |
| Coronary atherosclerosis and other forms of chronic ischemic heart disease | - | - | - | 1211 (10.3) | 1192 (10.1) | 0.01 | 836 (13.2) | 832 (13.1) | 0.00 | 1883 (11.9) | 1869 (11.8) | 0.00 | - | - |
| Other atherosclerosis | 39 (0.4) | 40 (0.4) | 0.00 | 1028 (8.7) | 1021 (8.7) | 0.00 | 797 (12.6) | 787 (12.4) | 0.00 | 899 (5.7) | 910 (5.8) | 0.00 | 2763 (6.3) | 2758 (6.3) |
| Any stroke | 344 (3.5) | 309 (3.2) | 0.02 | 560 (4.7) | 565 (4.8) | 0.00 | 238 (3.8) | 254 (4.0) | 0.01 | 877 (5.6) | 863 (5.5) | 0.00 | 2019 (4.6) | 1991 (4.6) |
| Ischemic stroke (w and w/o mention of cerebral infarction) | - | - | - | 473 (4.0) | 466 (3.9) | 0.00 | 210 (3.3) | 226 (3.6) | 0.01 | 761 (4.8) | 755 (4.8) | 0.00 | - | - |
| Hemorrhagic stroke | - | - | - | 94 (0.8) | 107 (0.9) | 0.01 | 27 (0.4) | 24 (0.4) | 0.01 | 118 (0.7) | 118 (0.7) | 0.00 | - | - |
| Other cerebrovascular disease | 30 (0.3) | 24 (0.2) | 0.01 | 118 (1.0) | 108 (0.9) | 0.01 | 36 (0.6) | 40 (0.6) | 0.01 | 133 (0.8) | 145 (0.9) | 0.01 | 317 (0.7) | 317 (0.7) |
| Late effects of cerebrovascular disease | 207 (2.1) | 197 (2.0) | 0.01 | 186 (1.6) | 184 (1.6) | 0.00 | 112 (1.8) | 117 (1.8) | 0.01 | 340 (2.2) | 340 (2.2) | 0.00 | 845 (1.9) | 838 (1.9) |
| CHF | 509 (5.2) | 487 (5.0) | 0.01 | 785 (6.7) | 767 (6.5) | 0.01 | 356 (5.6) | 349 (5.5) | 0.00 | 1212 (7.7) | 1202 (7.6) | 0.00 | 2862 (6.6) | 2805 (6.4) |
| Peripheral Vascular disease or surgery | 266 (2.7) | 256 (2.6) | 0.01 | 415 (3.5) | 403 (3.4) | 0.01 | 2028 (32.0) | 2023 (31.9) | 0.00 | 860 (5.4) | 866 (5.5) | 0.00 | 3569 (8.2) | 3548 (8.1) |
| Atrial fibrillation | 673 (6.9) | 617 (6.3) | 0.02 | 1166 (9.9) | 1149 (9.7) | 0.00 | 404 (6.4) | 414 (6.5) | 0.01 | 1465 (9.3) | 1472 (9.3) | 0.00 | 3708 (8.5) | 3652 (8.4) |
| Other cardiac dysrhythmia | 213 (2.2) | 200 (2.0) | 0.01 | 528 (4.5) | 525 (4.4) | 0.00 | 336 (5.3) | 342 (5.4) | 0.00 | 544 (3.4) | 560 (3.5) | 0.01 | 1621 (3.7) | 1627 (3.7) |
| Cardiac conduction disorders | 95 (1.0) | 89 (0.9) | 0.01 | 156 (1.3) | 142 (1.2) | 0.01 | 75 (1.2) | 73 (1.2) | 0.00 | 259 (1.6) | 248 (1.6) | 0.01 | 585 (1.3) | 552 (1.3) |
| Other CV disease | 452 (4.6) | 431 (4.4) | 0.01 | 395 (3.3) | 389 (3.3) | 0.00 | 200 (3.2) | 206 (3.2) | 0.01 | 710 (4.5) | 734 (4.6) | 0.01 | 1757 (4.0) | 1760 (4.0) |
| **Diabetes Medication Use** | | | | | | | | | | | | | | |
| **Number of antidiabetic substances at index date** | | | | | | | | | | | | | | |
| Mean | - | - | - | 2.1 | 2.1 | 0.03 | 2.2 | 2.2 | 0.01 | 2.1 | 2.2 | 0.02 | - | - |
| SD | - | - | - | 0.8 | 0.8 | - | 0.9 | 0.8 | - | 0.7 | 0.7 | - | - | - |
| Median | - | - | - | 2 | 2 | - | 2 | 2 | - | 2 | 2 | - | - | - |
| Q1; Q3 | - | - | - | 2.0; 2.0 | 2.0; 2.0 | - | 2.0; 3.0 | 2.0; 3.0 | - | 2.0; 3.0 | 2.0; 3.0 | - | - | - |
| Min; Max | - | - | - | 1.0; 7.0 | 1.0; 6.0 | - | 1.0; 7.0 | 1.0; 6.0 | - | 1.0; 6.0 | 1.0; 7.0 | - | - | - |
| Naïve new use of antidiabetic drugs at index date, N (%) | - | - | - | 184 (1.6) | 180 (1.5) | 0.00 | 179 (2.8) | 185 (2.9) | 0.01 | 74 (0.5) | 68 (0.4) | 0.01 | - | - |
| Initiation of the study drug (empagliflozin/any DPP-4i) as monotherapy, N (%) | - | - | - | 100 (0.8) | 90 (0.8) | 0.01 | 137 (2.2) | 150 (2.4) | 0.01 | 48 (0.3) | 44 (0.3) | 0.00 | - | - |
| Dual therapy of study drug with metformin (without the use of other antidiabetic drugs), N (%) | - | - | - | 7 (0.1) | 5 (0.0) | 0.01 | 334 (5.3) | 317 (5.0) | 0.01 | 721 (4.6) | 702 (4.4) | 0.01 | - | - |
| Sulfonylureas second generation (concomitant initiation or current use), N (%) | - | - | - | 25 (0.2) | 30 (0.3) | 0.01 | 1014 (16.0) | 1063 (16.8) | 0.02 | 1760 (11.1) | 1792 (11.4) | 0.01 | - | - |
| Insulin (concomitant initiation or current use), N (%) | - | - | - | 421 (3.6) | 438 (3.7) | 0.01 | 656 (10.3) | 664 (10.5) | 0.00 | 3817 (24.2) | 3911 (24.8) | 0.01 | - | - |
| CABG=coronary artery bypass grafting; CHF=congestive heart failure; CV=cardiovascular; DPP-4i=dipeptidyl peptidase-4 inhibitor; EMPA=empagliflozin; HbA1c=glycated hemoglobin; Max=maximum; MI=myocardial infarction; Min=minimum; PTCA=percutaneous transluminal coronary angioplasty; Q1=1st quartile; Q3=3rd quartile; SD=standard deviation; Std Diff=standardized difference; T2D=type 2 diabetes.  The combined comorbidity score is a single numeric comorbidity score for predicting short- and long-term mortality, by combining conditions in the Charlson and Elixhauser comorbidity measures. Presence of diagnosis codes indicating existence of certain conditions may themselves be indicators for other factors that are inversely associated with 1-year mortality or may reflect idiosyncrasies of administrative data, which is why negative values can occur. However, the underlying diagnoses should not be regarded as having a preventive/protective effect. JJ Gagne et al. *A combined comorbidity score predicted mortality in elderly patients better than existing scores.* J Clin Epidemiol. 2011 July;64(7):749–759. doi:10.1016/j.jclinepi.2010.10.004.  Look-back period for combined comorbidity score is ever before index date.  Look-back period for comorbidities and diabetes medication use is ever before index date. | | | | | | | | | | | | | | |
